# Small Extracellular Vesicle‐Derived Nicotinamide Phosphoribosyltransferase (NAMPT) Induces Acyl‐Coenzyme A Synthetase SLC27A4‐Mediated Glycolysis to Promote Hepatocellular Carcinoma

**DOI:** 10.1002/jev2.70071

**Published:** 2025-04-16

**Authors:** Cherlie Lot Sum Yeung, Tung Him Ng, Charlotte Jiaqi Lai, Tingmao Xue, Xiaowen Mao, Sze Keong Tey, Regina Cheuk Lam Lo, Chun‐Fung Sin, Kwan Ming Ng, Danny Ka Ho Wong, Lung‐Yi Mak, Man‐Fung Yuen, Irene Oi‐Lin Ng, Peihua Cao, Yi Gao, Jing Ping Yun, Judy Wai Ping Yam

**Affiliations:** ^1^ Department of Pathology, Li Ka Shing Faculty of Medicine The University of Hong Kong Hong Kong; ^2^ Department of Hepatobiliary Surgery II Zhujiang Hospital, Southern Medical University Guangzhou Guangdong China; ^3^ State Key Laboratory of Quality Research in Chinese Medicine, Institute of Chinese Medical Sciences University of Macau Macau; ^4^ Department of Surgery, School of Clinical Medicine, Li Ka Shing Faculty of Medicine The University of Hong Kong Hong Kong; ^5^ Laboratory for Synthetic Chemistry and Chemical Biology Limited, Hong Kong Science Park Hong Kong; ^6^ Department of Medicine, Li Ka Shing Faculty of Medicine The University of Hong Kong Hong Kong; ^7^ State Key Laboratory of Liver Research The University of Hong Kong Hong Kong; ^8^ Clinical Research Center, Zhujiang Hospital Southern Medical University Guangzhou Guangdong China; ^9^ Department of Pathology Sun Yat‐sen University Cancer Center Guangzhou Guangdong China; ^10^ Materials Innovation Institute for Life Sciences and Energy (MILES), HKU‐SIRI Shenzhen China

## Abstract

Tumour‐derived small extracellular vesicles (sEV) are critical mediators within the tumour microenvironment (TME) and are known to regulate various metabolic pathways. In metastatic hepatocellular carcinoma (HCC), mass spectrometry protein analysis of HCC‐derived sEV (HCC‐sEV) identified an upregulation of nicotinamide phosphoribosyltransferase (NAMPT), a key enzyme in maintaining cellular nicotinamide adenine dinucleotide (NAD+) levels. Our study demonstrates that sEV‐NAMPT enhances glycolysis, tumorigenesis, and metastasis in HCC. Specifically, sEV‐NAMPT activates the NF‐κB transcription factor through toll‐like receptor 4 (TLR4), leading to elevated SLC27A4 expression. SLC27A4 functions primarily as a long‐chain fatty acid transporter and acyl‐CoA synthetase. Lipidomic and metabolomic analyses revealed a positive correlation between SLC27A4 and intracellular levels of triacylglycerol (TG) and dihydroxyacetone phosphate (DHAP). Increased TG levels enhance lipolysis via hepatic lipase and facilitate the conversion of glycerol‐3‐P to DHAP, an intermediate that bridges lipid metabolism and glycolysis. This study uncovers a novel regulatory axis involving sEV‐NAMPT and SLC27A4 in glycolysis, independent of traditional fatty acid metabolism pathways. Clinically, targeting sEV‐NAMPT with the inhibitor FK866 significantly inhibited tumour growth in various HCC in vivo models, highlighting the potential of sEV‐NAMPT as both a biomarker and therapeutic target in HCC.

## Graphical abstract

NAMPT delivered by metastatic HCC cells‐derived sEV upregulates SLC27A4 expression via TLR4/NF‐кB dependent pathway. SLC27A4 acts as an acyl‐CoA synthetase and increases the level of intracellular TG, leading to DHAP‐mediated transition between lipogenesis and glycolysis. The enhanced glycolytic activity leads to HCC tumorigenesis and metastasis.

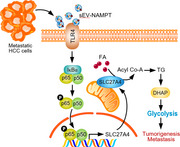

## 1 Introduction

Tumour development involves intricate multistep processes, with cellular metabolic reprogramming emerging as a major deregulated hallmark (DeBerardinis and Chandel 2016). Sustaining an adequate energy supply is critical for cancer cells as they constantly proliferate and strive to survive in hostile conditions. The well‐known Warburg effect revealed that cancer cells prefer aerobic glycolysis to oxidative phosphorylation (Vander Heiden et al. 2009), even when oxygen availability is sufficient. This aberrant metabolic pattern correlates with more aggressive tumorigenic features and a poorer prognosis (Fernández et al. 2020; Liberti and Locasale 2016). Studies have shown that substrates produced during glycolysis, ranging from glycolytic intermediates to enzymes, are important in shaping a conducive tumour microenvironment (TME) (Zhou et al. 2022). For instance, it has been demonstrated that lactic acid, a byproduct of glycolysis, favours the formation of an immunosuppressive TME, thereby impairing the efficacy of immunotherapy (Kumagai et al. 2022). Glycolytic metabolites have been implicated in angiogenesis and metastasis as well (Kes et al. 2020). Given the phenotypic commonality of glycolysis in multiple cancers, a comprehensive understanding of its contributing factors is imperative for developing suitable therapeutic interventions.

Hepatocellular carcinoma (HCC) is the most common form of liver cancer, which originates from hepatocytes and contributes to more than 80% of the diagnosed cases (Lazarevich et al. 2004). An enhanced glycolytic rate, which promotes tumour growth and survival, is commonly observed in HCC. The rapid production of ATP, metabolite intermediates, and subsequent glutaminolysis is beneficial for HCC progression (Ganapathy‐Kanniappan 2018; Xu and Herschman 2019). Furthermore, the continuous production of lactic acid by glycolysis assists in the formation of an acidified TME, which promotes ECM degradation and metastasis (Schornack and Gillies 2003). It also triggers the release of angiogenic factors such as vascular endothelial growth factor and plasminogen activator inhibitor‐1, further promoting vascular supply (Yasuda et al. 2004; Zhang et al. 2021). Although glycolysis has been extensively studied in cancer for several years, the mechanism by which cancer cells adopt glycolytic hyperactivity remains unclear. Some studies have suggested that this metabolic switch occurs during the early stages of cancer development, during which cells harbouring glycolytic mutations are more likely to survive and confer a selective advantage by passing on these oncogenes (Gatenby and Vincent 2003). Others have proposed that enhanced glycolysis is a consequence of cancer cells adapting to unfavourable hypoxic environments (Pelletier et al. 2012).

Small extracellular vesicles (sEV) are lipid membrane‐enclosed nanoparticles that range in size from 40 to 150 nm (Jeppesen et al. 2019). They are present in almost all biological fluids (Théry et al. 2006) and play a crucial role in intercellular communication as carriers of diverse biological cargo, including nucleic acids, proteins, and lipids (Abhange et al. 2021). The biogenesis of sEV is a dynamic and highly coordinated process that initiates with inward budding of the cell membrane to form multivesicular bodies (Bobrie et al. 2011). Upon maturation, sEV is released into the extracellular space and delivered to targeted recipient cells via surface antigen presentation and chemo‐attractiveness (Arima et al. 2019; Basak et al. 2022). Notably, cancer cells use sEV for communication. Several studies have reported that tumour‐derived sEV can promote cancer growth and metastasis (Kok and Yu 2020).

In the present study, we provide insights into the role of sEV in the modulation of HCC metabolism. Our findings demonstrated that sEV derived from metastatic HCC cells delivered the nicotinamide phosphoribosyltransferase (NAMPT). Importantly, we established that sEV‐NAMPT promotes tumorigenesis and metastasis and accelerates glycolysis in recipient cells. Additionally, our investigation revealed that the functional effect of sEV‐NAMPT is regulated by SLC27A4. We found that sEV‐NAMPT induced‐SLC27A4 overexpression via the NF‐κB signalling pathway, and this activation is dependent on Toll‐like receptor 4 (TLR4). Intriguingly, our study also demonstrated that SLC27A4 mainly functions as an acyl‐CoA synthetase in HCC rather than a conventional fatty acid transporter. Hence, the production of fatty acyl‐CoA by SLC27A4 enhances intercellular hepatic triacylglycerol (TG) levels. SLC27A4 correlated with dihydroxyacetone phosphate (DHAP), which facilitates the transition from lipogenesis to glycolysis. From a clinical perspective, our results demonstrated that NAMPT levels were elevated in the circulating sEV of HCC patients compared to those in control individuals and patients with hepatitis B virus (HBV) infection and cirrhosis. Furthermore, we observed a reduction in sEV‐NAMPT levels in most patients after tumour resection, underscoring the clinical significance of sEV‐NAMPT in HCC. Finally, we provide evidence of the effectiveness of an inhibitor, FK866, in blocking the functional activity of sEV‐NAMPT, suggesting its potential as a therapeutic target in HCC.

## 2 Materials and Methods

### 2.1 Clinical Samples

Fifty‐one pairs of primary tumour tissues and matched adjacent non‐tumorous tissues were resected from patients with HCC at the Queen Mary Hospital, Hong Kong. RNA was isolated from the specimens for NAMPT expression analysis. Blood samples were collected from individuals without liver disease (control subjects) and from patients with HCC who had not received any treatment. Blood samples were collected at Queen Mary Hospital, Hong Kong, and Zhujiang Hospital, Guangzhou, China. Detailed information on the blood donors is provided in Table S1. Circulating sEV was isolated from blood samples to measure the serum sEV‐derived NAMPT levels. A tissue microarray was constructed using paired HCC specimens and adjacent non‐tumorous tissues obtained from the archives of the Department of Pathology, Sun Yat‐sen University Cancer Center (SYSUCC). The tissue microarray was used for immunohistochemical analysis. The use of human samples was approved by the Institutional Review Board of the University of Hong Kong/Hospital Authority Hong Kong West Cluster (HKU/HA HKW IRB) (protocol numbers: UW 11–448 and UW 17–22), Zhujiang Hospital of Southern Medical University (protocol number: 2017‐GDEK‐003), and the Institute Research Medical Ethics Committee of SYSUCC. Written informed consent was obtained from all the patients.

### 2.2 Cell Culture

Hep3B, PLC/PRF/5, Huh7 and 293FT cells were purchased from American Type Culture Collection. HLE cells were given by the Japanese Collection of Research Bioresources. MHCC97L and MHCCLM3 cells were obtained from the Cancer Institute,  Fudan University, China (Sun et al. 1996). MIHA cells were provided by Jayanta Roy‐Chowdhury, Albert Einstein College of Medicine, New York (Brown et al. 2000). Murine p53‐null hepatoblasts transduced by Myc (p53−/−;Myc hepatoblasts) were provided by Scott Lowe, Memorial Sloan Kettering Cancer Center, New York (Xue et al. 2012). All cells were cultured in high‐glucose Dulbecco's Modified Eagle Medium supplemented with 10% fetal bovine serum (FBS) and maintained at 37°C in a humidified incubator containing 5% CO_2_. All cells were authenticated by short tandem repeat profiling and confirmed mycoplasma‐free.

### 2.3 Expression Constructs and Stable Cell Lines

Short hairpin RNA (shRNA) oligos targeting NAMPT (shNAMPT), SLC27A4 (shSLC27A4) and TLR4 (shTLR4), and single guide RNA oligos inducing expression of NAMPT (sgNAMPT) were synthesized by Integrated DNA Technologies. Sequences of oligos were listed in Table S2. The shRNA duplexes were subcloned into MISSION pLKO.1‐puro empty vector (Sigma–Aldrich) via AgeI and EcoRI sites. The sgRNA duplexes were subcloned into lenti sgRNA(MS2)_zeo backbone via BsmBI sites. The plasmids were transformed into bacteria and amplified. The incorporation of correct inserts was confirmed by DNA sequencing. To establish stable cell lines, plasmids were co‐transfected with HIV packaging mix into 293FT cells using EndoFectin Lenti transfection reagent (GeneCopoeia). The lentivirus produced was used to infect cells using polybrene as an infection reagent. Infected cells were selected with respective antibiotics. NAMPT‐knockdown (NAMPT‐KD1 and NAMPT‐KD2) cells were established in MHCC97L and MHCCLM3 cells using shNAMPT plasmids. SLC27A4‐knockdown (SLC27A4‐KD1 and SLC27A4‐KD2) and TLR4‐knockdown (TLR4‐KD1 and TLR4‐KD2) cells were established in PLC/PRF/5 and HLE cells using shSLC27A4 and shTLR4 plasmids, respectively. Respective non‐target knockdown control (CTL‐KD) cells were generated using MISSION non‐target shRNA control vector (Sigma–Aldrich). NAMPT overexpressing cells (NAMPT‐SAM1 and NAMPT‐SAM3) were established in HLE cells using sgNAMPT following CRISPR/gRNA‐directed synergistic activation mediator method (Konermann et al. 2015). Non‐target sgRNA was used to establish respective control (CTL‐SAM). Expressions in infected cells were validated by Western blotting analysis.

### 2.4 Isolation of sEV

#### 2.4.1 From Cultured Conditioned Medium

The collection of sEV from culture medium has been described in our previous studies with slight modifications (Tey et al. 2022). To collect sEV from cells, cells were seeded on petri dish at around 70% confluency. After 24 h, when cells were attached, they were washed twice with PBS and replenished with culture medium (DMEM‐HG) supplemented with 10% sEV‐depleted FBS. sEV‐depleted FBS was previously prepared by centrifugation at 100,000×*g* for more than 16 h (4°C) using SW45Ti Fixed‐Angle rotor (Beckman Coulter). The supernatant FBS was collected without disturbing the centrifugated pellet. After 72‐h culturing in sEV‐depleted FBS supplemented medium, or when cells reached maximum confluency, the conditioned medium was collected and sEVs were isolated by differential centrifugation using SW45Ti Fixed‐Angle rotor (Beckman Coulter). In brief, conditioned medium was centrifuged at 3,000×*g* for 15 min at 4°C to remove dead cells and cell debris. Then, the conditioned medium was added and topped up in 70 mL polycarbonate bottle with cap assembly. After balancing the tubes, they were centrifuged at 20,000×*g* for 30 min at 4°C. The supernatant was then filtered through 0.22 µm filter to remove microvesicles. The resulting medium was centrifuged at 100,000×*g* for 70 min at 4°C to pellet sEV. The sEV pellet were washed with PBS and further centrifuged at 100,000×*g* for 70 min at 4°C. Pelleted sEV were finally resuspended in PBS.

#### 2.4.2 From Serum Samples

The collection of sEV from serum samples has been described in our previous studies (Tey et al. 2022; Xu et al. 2023). The whole blood was placed in room temperature for 30 min, allowing the formation of blood clot. The blood sample was then centrifuged at 1,500×*g* for 10 min at 4°C, and the supernatant (serum) was slowly extracted without disturbing the clot. Serum (1 mL) was then topped up to 70 mL by PBS and subjected to ultracentrifugation using SW45Ti Fixed‐Angle rotor (Beckman Coulter) at 20,000×*g* for 30 min at 4°C. The supernatant was then filtered through 0.22 µm filter, followed by ultracentrifugation at 100,000×*g* for 2 h at 4°C. Finally, the sEV pellet was washed by another round of ultracentrifugation at 100,000×*g* for 2 h at 4°C by PBS, of which the sEV pellet was resuspended using PBS and stored at −80°C. The sEV collected from control individuals, patients with hepatitis B infection, cirrhosis and HCC were validated for their expressions of Alix, TSG101, CD9 and GM130 by Western blotting analysis. The size range of sEV was determined by nanoparticle tracking analysis and sEV were subjected to immunogold labelling to reveal the presence of CD63. The results of validation have been shown in the supplementary data of our previous publication (Tey et al. 2022).

### 2.5 Characterization of sEV

Isolated sEV were characterized by nanoparticle tracking analysis, observed under electron microscope upon immunogold labelling, and examined for positive (TSG101 and CD9) and negative (p62 and GM130) sEV marker by Western blotting analysis as detailed below.

### 2.6 Nanoparticle Tracking Analysis

The size of sEV was determined by ZetaView TWIN NTA PMX‐220 (Particles Metrix GmbH). Briefly, 1 mL of sEV (diluted at around 0.1 ng/mL using Milli‐Q Type 1 Ultrapure Water) was injected into the sample cell. Eleven distinct positions were measured under standard instrument settings (sensitivity: 75; shutter: 100; brightness: 30; min. area: 10; max area: 1000; fps: 30; temperature: 25°C). Peak analysis was performed by ZetaView software (version 8.05.11).

### 2.7 Examination of sEV by Transmission Electron Microscopy (TEM)

#### 2.7.1 Negative Staining of sEV

Fresh samples without subjected to freezing were diluted to 0.1 µg/µL and fixed in 2% 0.22 µm‐filtered formaldehyde. Samples (10 µL) were then added onto discharged copper electron microscope grids, allowing absorption onto the carbon film for 10 min. The grids were dried gently on a filter paper and added onto 2% uranyl acetate for 1 min. The grid was then washed with water and allowed to dry on a filter paper in a petri dish. Imaging was performed on Tecnai G2 20 Scanning TEM and captured by ORIUS SC600 Model CCD Camera with Gatan digital micrograph software.

#### 2.7.2 Immunogold Labelling of sEV

Isolated sEV were resuspended in PBS and allowed to absorb on Formvar‐carbon coated electron microscope grids for 20 min. Then, the grids were blocked in 1% bovine serum albumin (BSA) in PBS and incubated with anti‐CD63 antibody for 30 min, followed by 10 nm gold‐conjugated secondary antibodies for 20 min. The grids were then washed with PBS, incubated with 1% glutaraldehyde, contrasted, embedded and observed under Philips CM100 Transmission Electron Microscope.

#### 2.7.3 In Vivo Therapeutic Effect of FK866 and Sorafenib Co‐Treatment

Five‐week‐old male mice were implanted with patient‐derived xenograft as established elsewhere (Lo et al. 2016). When xenograft became palpable, mice were randomly assigned to one of four treatment groups: (1) vehicle, (2) FK866, (3) sorafenib and (4) FK866 + sorafenib. FK866 at 5 mg/kg mouse body weight was injected subcutaneously beside tumour every 3 days. Sorafenib at 30 mg/kg mouse body weight was administered via oral gavage every day. Mice body weight and tumour growth were monitored throughout the experiment. After 21 days of treatment, mice were sacrificed. Tumours were excised, weighed and examined.

### 2.8 Statistical Analysis

All statistical tests were performed using the GraphPad Prism 9 software. Student's *t*‐test was used to compare the means between the two groups. One‐way analysis of variance (ANOVA) was used to compare the means between more than two groups. Two‐way ANOVA was used for the time‐course analysis. The Bonferroni correction was used to adjust for multiple comparisons. Data are expressed as mean ± standard error of the mean (SEM) from three independent experiments. A two‐sided *p* value less than 0.05 was considered statistically significant.

## 3 Results

### 3.1 Metastatic HCC Cell‐Derived sEV Drive Oncogenic and Glycolytic Properties

**FIGURE 1:**
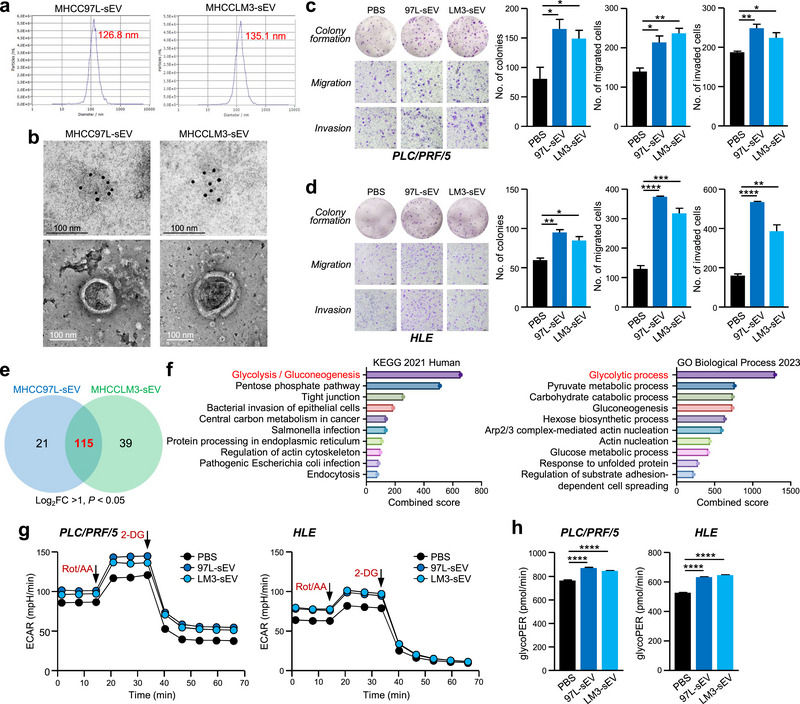
**Metastatic HCC cell‐derived sEVs facilitate HCC cell glycolysis and promote the cancerous properties of recipient cells. (a)** The size distribution of sEV derived from MHCC97L and MHCCLM3 cells was measured using a nanoparticle tracking analyser. **(b)** Representative electron micrographs of sEV subjected to immunogold labelling using anti‐CD63 antibodies followed by secondary antibodies coupled to 10‐nm gold particles (*upper panel*). Scale bar, 100 nm; magnification, 52,000×. sEV morphology was shown by negative staining (*lower panel*). PLC/PRF/5 **(c)** and HLE **(d)** cells treated with the indicated sEV were subjected to colony formation, migration and invasion assays. Representative images of colonies and cells are shown. The numbers of colonies and cells were quantified. **(e and f)** sEV from MHCC97L, MHCCLM3 and MIHA cells were analysed via proteomics mass spectrometry analysis. The upregulated proteins in MHCC97L‐ and MHCCLM3‐sEV compared to those in MIHA‐sEV (fold change > 2, *p* < 0.05) were identified. **(e)** Venn diagram showing upregulated proteins in MHCC97L‐ and MHCCLM3‐sEV. **(f)** Pathway analysis of the commonly upregulated proteins using Gene Ontology (GO) biological process and Kyoto Encyclopedia of Genes and Genomes (KEGG) databases. **(g)** Seahorse glycolytic rate assay was used to monitor real‐time changes in the ECAR of PLC/PRF/5 and HLE cells treated with the indicated sEV. The additions of rotenone/antimycin A (Rot/AA) and 2‐deoxyglucose (2‐DG) are indicated. **(h)** The measured glycoPER is shown. The data are expressed as the mean ± SEM. ****p < 0.0001, ***p < 0.001, **p < 0.01, *p < 0.05.

Tumour‐derived sEV play important roles as mediators of the TME. To explore the effect of tumour‐derived sEV on HCC, we isolated sEV from the conditioned medium of two metastatic HCC cell lines (MHCC97L and MHCCLM3) by differential ultracentrifugation. The isolated sEV were verified by ZetaView nanoparticle analysis and transmission electron microscopy (Figure 1a,b). Treatment with these metastatic HCC‐sEVs promoted the migration, invasion, and colony formation of non‐metastatic HCC cells (PLC/PRF/5 and HLE) (Figure 1c,d). To determine the underlying mechanism that promotes HCC oncogenesis, mass spectrometry (MS) protein analysis was performed on 97L‐sEV, LM3‐sEV and sEV from the normal liver cell line MIHA (MIHA‐sEV). Compared with MIHA‐sEVs, the expression of proteins in both 97L‐sEV and LM3‐sEV was significantly upregulated, with 137 upregulated proteins in 97L‐sEV and 154 upregulated proteins in LM3‐sEV, reflecting at least a two‐fold increase in expression. Among the upregulated proteins, 115 were unanimously overexpressed (Figure 1e). Analysis of these proteins using Gene Ontology (GO) enrichment analysis and Kyoto Encyclopedia of Genes and Genomes (KEGG) analysis revealed that glycolysis was strongly enhanced (Figure 1f). Based on these results, the metabolic properties of the 97L‐sEV and LM3‐sEV were tested. Treatment of target cells with metastatic HCC‐sEVs resulted in an increase in glycolysis, as evidenced by an increase in the extracellular acidification rate (ECAR) and glycolytic proton efflux rate (glycoPER) (Figure 1g,h), thus confirming the ability of metastatic HCC‐sEVs to control glycolysis. These findings collectively suggest that metastatic HCC‐sEVs mediate glycolysis and support liver tumour development.

### 3.2 Elevated NAMPT Levels in Metastatic HCC Cell Line‐Derived sEV

**FIGURE 2:**
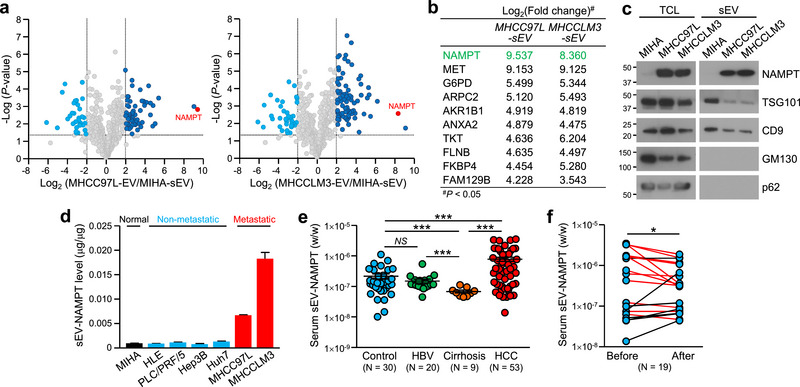
**Upregulation of NAMPT in the sEV of metastatic HCC cells and circulating sEV of HCC patients. (a)** Volcano plots depicting the proteins differentially expressed between MHCC97L‐sEV (*left*) and MHCCLM3‐sEV (*right*) versus MIHA‐sEV. (**b)** The top 10 upregulated proteins identified in MHCC97L‐sEV, ranked by fold change, are presented. The corresponding fold changes in expression in MHCCLM3‐sEV are listed. **(c)** Immunoblotting of NAMPT, positive sEV markers (TSG101 and CD9) and negative sEV markers (*cis*‐Golgi marker GM130 and nucleoporin p62) in total cell lysates (TCLs) and sEV. **(d)** Analysis of NAMPT levels in sEV derived from cell lines using ELISA. **(e)** Analysis of the NAMPT level in circulating sEV from control individuals (*n* = 30) and patients with HBV (*n* = 20), cirrhosis (*n* = 9) or HCC (*n* = 53) using ELISA. **(f)** Analysis of NAMPT levels in circulating sEV from HCC patients collected before and after surgery (*n* = 19). The data are presented as the means ± SEMs. ***p < 0.001. *NS*, not significant.

Considering the role of metastatic HCC‐sEVs in the promotion of glycolysis and tumorigenesis, we investigated the critical protein components responsible for these effects. Notably, the expression of NAMPT, a top‐ranked protein, was significantly elevated in both 97L‐sEV and LM3‐sEV to MIHA‐sEVs (Figure 2a,b). NAMPT is a critical rate‐limiting enzyme that regulates the catalytic conversion of nicotinamide to nicotinamide mononucleotide (NMN) to regenerate nicotinamide adenine dinucleotide (NAD+) (Ju et al. 2016). Abnormal NAMPT levels have been reported to regulate glycolysis in multiple cancers, including glioblastoma and pancreatic cancer (Tateishi et al. 2016). Therefore, we were interested in the role of NAMPT in sEV and its contribution to HCC carcinogenesis and glycolysis. To validate our MS results, the overexpression of NAMPT in metastatic HCC‐sEVs was confirmed (Figure 2c). Moreover, sEV‐NAMPT levels were positively correlated with the metastatic potential of the HCC cells (Figure 2d). We further compared the levels of NAMPT in circulating sEV from non‐HCC control subjects to those in patients with HBV, cirrhosis, and HCC. Our clinical data showed that sEV‐NAMPT levels were significantly higher in patients with HCC than in controls and in patients with HBV and cirrhosis (Figure 2e). After tumour resection, 12 of the 19 patients (approximately 63%) had reduced sEV‐NAMPT levels (Figure 2f). These data coincide with those of our mass spectrometry analysis, highlighting the association between elevated sEV‐NAMPT levels and HCC progression.

### 3.3 sEV‐NAMPT Is Responsible for the Promoting Effect of HCC‐sEV on Glycolysis and Carcinogenesis

**FIGURE 3:**
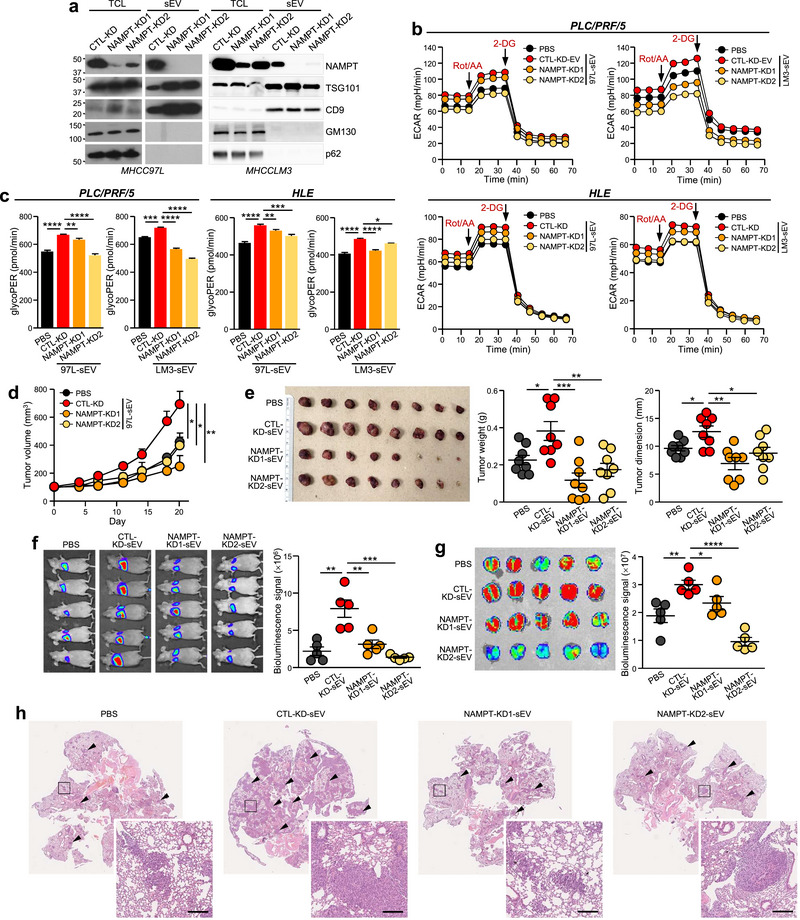
**Knockdown of NAMPT in sEV derived from metastatic HCC cells reduces the promoting effect on glycolysis and cancer phenotypes in recipient cells. (a)** Immunoblotting of NAMPT, sEV‐positive and sEV‐negative markers in total cell lysate (TCL) and sEV from CTL‐KD and NAMPT‐KD cells established from MHCC97L and MHCCLM3 cells. **(b)** Seahorse glycolytic rate assay was used to monitor real‐time changes in the ECAR of PLC/PRF/5 and HLE cells treated with the indicated sEV. The additions of rotenone/antimycin A (Rot/AA) and 2‐deoxyglucose (2‐DG) are indicated. **(c)** The measured glycoPER is shown. **(d)** PLC/PRF/5 cells were subcutaneously co‐injected with the indicated sEV into mice. The tumour volume was monitored twice per week. **(e)** Photograph of excised tumours is shown. Tumour weights and dimensions were measured. **(f)** Mice were intravenously injected with murine p53−/−;Myc hepatoblasts and the indicated sEV. Two weeks after injection, bioluminescence imaging was performed. **(g)** Ex vivo bioluminescence imaging of excised lung tissues. The luciferase signal was quantified. **(h)** Representative H&E‐stained micrographs showing tumour nodules in the lungs (indicated by arrowheads). Inlets show enlarged images. Scale bar, 200 µm. The data are expressed as the mean ± SEM. ****p < 0.0001, ***p < 0.001, **p < 0.01, *p < 0.05.

To explore the metabolic functionality of sEV‐NAMPT, NAMPT expression was suppressed in metastatic MHCC97L and MHCCLM3 cells (NAMPT‐KD1 and NAMPT‐KD2), with non‐targeting stable cells serving as controls (CTL‐KD). We observed that a reduction in the cellular level of NAMPT led to a corresponding decrease in the level of NAMPT in isolated sEV (Figures 3a and S1a,b). Consistent with our previous findings, the treatment of PLC/PRF/5 and HLE cells with CTL‐KD‐sEVs resulted in an augmented glycolytic rate and oncogenesis, whereas NAMPT‐KD‐sEVs were less able to trigger such promoting effects (Figures 3b,c and S2a–d). To determine the specific functions of sEV‐NAMPT, we overexpressed NAMPT (NAMPT‐SAM1 and NAMPT‐SAM3) in HLE cells using an empty vector (CTL‐SAM) as a control. Increased expression of cellular NAMPT resulted in an increase in the level of NAMPT in secreted sEV (Figure S3a–c). In agreement with our expectations, treatment of recipient cells with NAMPT‐SAM‐sEVs resulted in an increase in the glycolytic rate and oncogenesis, whereas such increases were not observed in response to treatment with CTL‐SAM‐sEVs (Figure S4a–e).

To further investigate the impact of sEV‐NAMPT in vivo, PLC/PRF/5 cells were subcutaneously injected into immunodeficient mice co‐treated with different sEVs. Compared with those in the PBS group, the tumours were substantially larger when CTL‐sEVs were added. However, this effect was not observed in the mice treated with NAMPT‐KD‐sEVs (Figure 3d,e). Extrahepatic metastasis to the lung is commonly observed in late‐stage HCC and is correlated with poor clinical outcomes. Therefore, we examined the effect of sEV on HCC metastasis. Luciferase‐labelled murine p53−/−;Myc hepatoblasts were co‐injected with PBS, CTL‐KD‐sEVs or NAMPT‐KD‐sEVs into nude mice, after which the lungs were colonized for 2 weeks. Notably, lung colonization ability and the number of metastatic lesions were markedly greater in the CTL‐KD‐sEV treatment group than in the PBS and NAMPT‐KD‐sEV groups (Figure 3f–h), suggesting that sEV‐NAMPT contributes to HCC tumorigenesis and metastasis. Notably, the in vivo oncogenic potential of the sEV was exacerbated when NAMPT was overexpressed. Co‐injection of NAMPT‐SAM‐sEVs also resulted in accelerated tumour development, leading to the formation of larger subcutaneous tumours than those in the PBS and CTL‐SAM groups (Figure S5a, b). A similar trend was also observed in the lung colonization model, in which more metastatic lesions were observed in the groups treated with NAMPT‐SAM‐sEVs (Figure S5c–e). Taken together, these data demonstrated the oncogenic role of sEV‐NAMPT in promoting glycolysis and tumorigenesis in HCC.

### 3.4 sEV‐NAMPT Regulates SLC27A4 Through TLR4‐Dependent NF‐κB Activation

**FIGURE 4:**
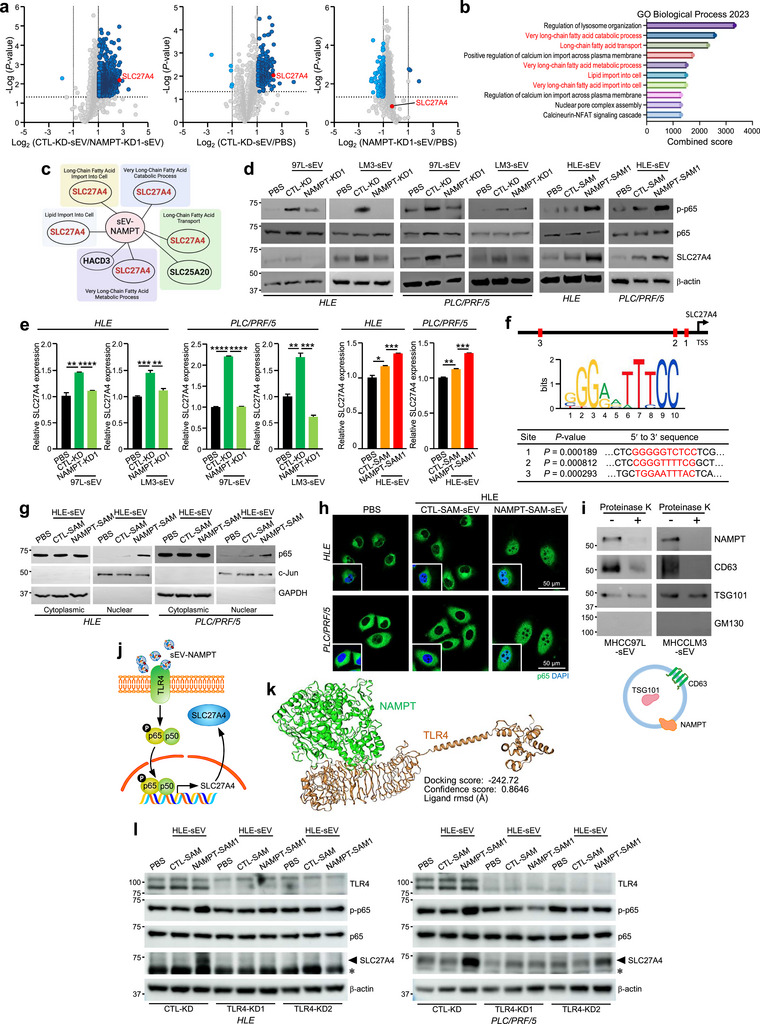
**sEV‐NAMPT regulates SLC27A4 through TLR4‐dependent NF‐κB activation. (a)** HLE cells were treated with PBS, CTL‐KD‐sEV or NAMPT‐KD‐sEV derived from MHCC97L cells and subjected to proteomic analysis. Volcano plots depicting the differentially expressed proteins (DEPs) in cells treated with CTL‐KD‐sEV versus NAMPT‐KD1‐sEV *(left)*, CTL‐KD‐sEV versus PBS (*middle*), and NAMPT‐KD1‐sEV versus PBS *(right)*. **(b)** Gene Ontology (GO) analysis of the upregulated proteins (fold change > 2, *p* < 0.05). Biological processes related to lipid metabolism were identified and are highlighted in red. **(c)** The proteins involved in the identified processes in the GO analysis are listed. **(d)** Immunoblotting of p‐p65, total p65 and SLC27A4 in HLE and PLC/PRF/5 cells treated with the indicated sEV. β‐Actin was used as an internal control. **(e)** Quantitative PCR analysis of SLC27A4 levels in cells subjected to the same treatment as described in (d). **(f)** Illustration showing three putative NF‐κB binding sites in the promoter region upstream of the SLC27A4 transcription start site (TSS). The NF‐κB binding motif, interacting nucleotides (red) and binding significance are shown. **(g)** Immunoblotting of p65 in the cytoplasmic and nuclear fractions of cells upon sEV treatment. c‐Jun and GAPDH were used as nuclear and cytoplasmic markers, respectively. **(h)** Representative immunofluorescence images of p65 (green) in cells treated with the indicated sEV are shown. Nuclei were counterstained with DAPI (blue) and are shown in the inserts. Scale bar, 50 µm. **(i)** Immunoblotting of NAMPT and sEV marker expression in sEV treated with or without proteinase K (*upper panel*). The localization of NAMPT and sEV markers is illustrated in the diagram (*lower panel*). **(j)** A schematic diagram depicting the proposed pathway through which sEV‐NAMPT activates NF‐κB‐mediated SLC27A4 transcription through TLR4. **(k)** Binding analysis of NAMPT and TLR4 using HDOCK. **(l)** Immunoblotting of TLR4, p‐p65, total p65 and SLC27A4 (indicated by arrowhead) in CTL‐KD and TLR4‐KD cells treated with the indicated sEV. A non‐specific band is marked by an asterisk. β‐Actin was used as an internal control. The data are expressed as the mean ± SEM. ****p < 0.0001, ***p < 0.001, **p < 0.01, *p < 0.05.

The oncogenic and glycolytic effects of sEV‐NAMPT prompted us to investigate its regulatory mechanism in recipient cells. To investigate this phenomenon, we conducted proteomic profiling of HLE cells treated with PBS, CTL‐KD‐sEVs or NAMPT‐KD‐sEVs derived from MHCC97L cells (Figure 4a). We then performed GO enrichment analysis of the top 10 proteins that were upregulated upon CTL‐KD‐sEV activation but downregulated after NAMPT‐KD‐sEV treatment (Table S3). Contrary to our expectations, lipid metabolism pathways were mainly activated in sEV‐NAMPT‐treated cells, but not in glycolytic pathways (Figure 4b). Among these genes, solute carrier family 27 member 4 (SLC27A4), a fatty acid transporter, is involved in multiple pathways (Figure 4c). Remarkably, both the protein and transcriptional levels of SLC27A4 increased in HLE and PLC/PRF/5 cells upon sEV‐NAMPT treatment (Figure 4d,e). In silico analysis identified NF‐κB consensus‐binding motifs within 2 kb of the SLC27A4 promoter region (Figure 4f). Immunoblotting revealed NF‐κB activation, characterized by increased Ser‐536 phosphorylation of p65 (Figure 4d) and increased nuclear p65 levels in recipient cells following sEV‐NAMPT treatment (Figure 4g). Immunofluorescence staining further confirmed the induction of p65 nuclear translocation by sEV‐NAMPT (Figure 4h).

Previous reports have indicated that extracellular NAMPT (eNAMPT) shares a remarkable structural similarity with the TLR4‐binding protein, suggesting its ability to activate NF‐κB through direct ligation to TLR4 (Camp et al. 2015). Considering the localization of NAMPT on the surface of metastatic sEV (Figure 4i), we hypothesized that sEV‐NAMPT might exhibit a similar binding affinity to TLR4, thereby initiating NF‐κB‐mediated upregulation of SLC27A4 (Figure 4j). Indeed, in silico analysis predicted high‐affinity binding between NAMPT and TLR4 (Figure 4k). Moreover, the induction Ser‐536 p65 phosphorylation and overexpression of SLC27A4 by sEV‐NAMPT were less prominent in TLR4‐KD cells (Figure 4l), as compared to CTL‐KD cells, confirming the partial dependence of the sEV‐NAMPT‐mediated NF‐κB‐SLC27A4 axis on TLR4.

### 3.5 The Promoting Effect of sEV‐NAMPT Was Reduced in TLR4‐Knockdown Cells

**FIGURE 5:**
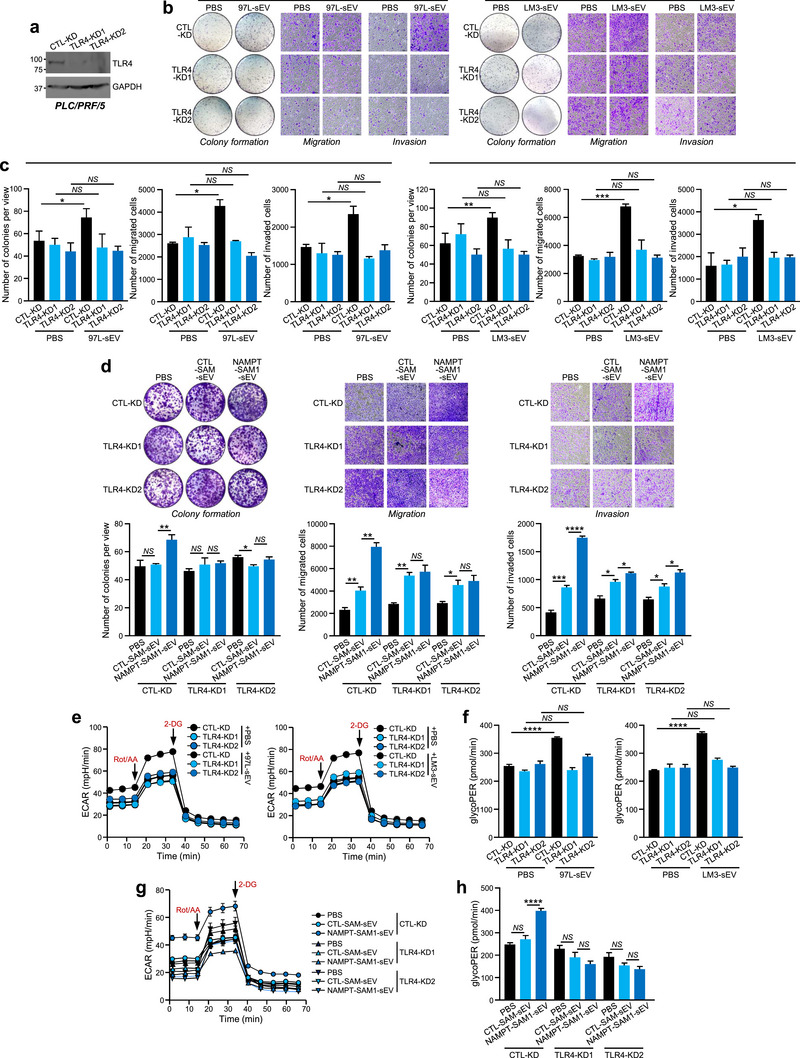
**Knockdown of TLR4 reduces the responsiveness of cells to the oncogenic activity of sEV‐NAMPT. (a)** Immunoblotting of TLR4 in CTL‐KD and TLR4‐KD PLC/PRF/5 cells. **(b)** PLC/PRF/5 CTL‐KD and TLR4‐KD cells treated with sEV derived from metastatic HCC cells (97L‐sEV and LM3‐sEV) were subjected to colony formation, migration and invasion assays. Representative images of colonies and cells are shown. **(c)** The numbers of colonies and cells were quantified. **(d)** PLC/PRF/5 CTL‐KD and TLR4‐KD cells treated with PBS, CTL‐SAM‐sEV or NAMPT‐SAM‐sEV were subjected to in vitro functional assays. (**e)** Analysis of the ECAR of PLC/PRF/5 CTL‐KD and TLR4‐KD cells treated with sEV derived from metastatic HCC cells (97L‐sEV and LM3‐sEV). (**f)** The measured glycoPER is shown. (**g)** PLC/PRF/5 CTL‐KD and TLR4‐KD cells treated with PBS, CTL‐SAM‐sEV or NAMPT‐SAM‐sEV were subjected to an ECAR assay. (**h)** GlycoPER was measured. The additions of rotenone/antimycin A (Rot/AA) and 2‐deoxyglucose (2‐DG) are indicated. The data are expressed as the mean ± SEM. ****p < 0.0001, ***p < 0.001, **p < 0.01, *p < 0.05. *NS*, not significant.

To extend our study further, we analysed the functional changes in TLR4‐KD cells in response to sEV‐NAMPT treatment and found similar changes in CTL‐KD cells. Notably, reduction in TLR4 levels did not significantly affect the intrinsic cancer properties of PLC/PRF/5 or HLE cells, as evidenced by the negligible differences observed in migration, invasion, and colony formation ability. However, disruption of TLR4 expression consistently impaired the responsiveness of recipient cells to sEV derived from MHCC97L and MHCCLM3 cells (Figures 5a–c and S6a–c). Specifically, the stimulatory effect of NAMPT‐SAM‐sEVs on the cancerous properties of recipient cells was abolished in TLR4‐KD cells (Figures 5d and S6d). Furthermore, the increase in glycolysis induced by sEV derived from MHCC97L and MHCCLM3 cells was significantly attenuated in TLR4‐KD cells compared to that in CTL‐KD cells (Figure 5e,f and S6e,f). The insensitivity of TLR4‐KD cells to NAMPT‐SAM‐sEV‐induced glycolysis further indicated the reliance of sEV‐NAMPT on TLR4 expression (Figures 5g,h and S6g,h).

### 3.6 SLC27A4 is the Downstream Target of sEV‐NAMPT That Promotes HCC Aggressiveness

**FIGURE 6:**
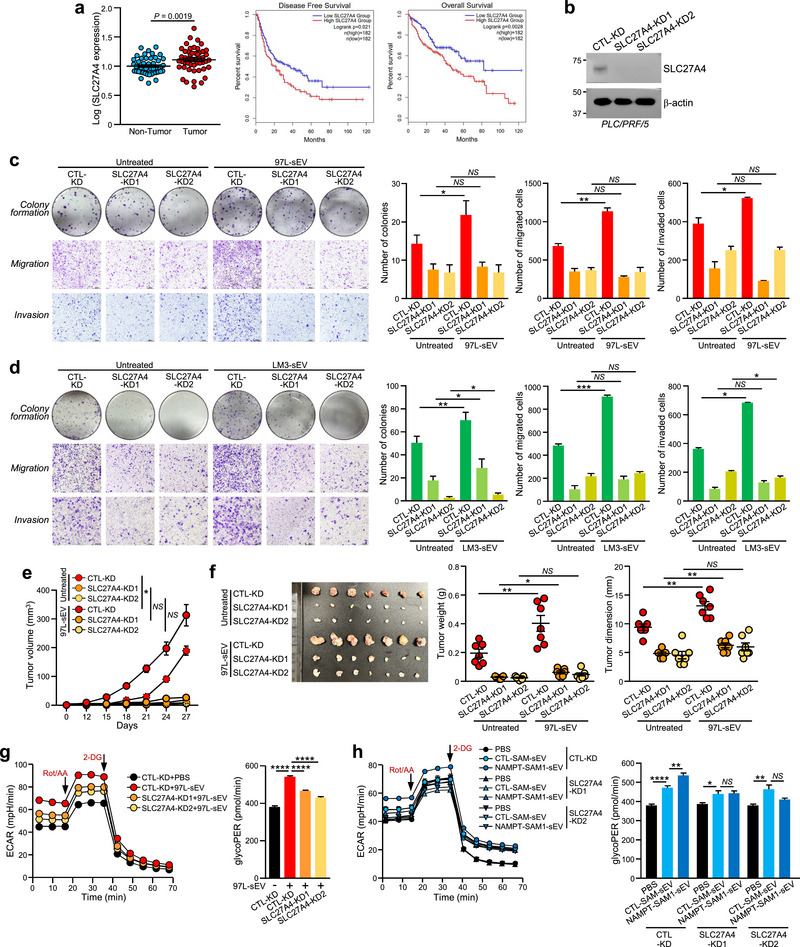
**SLC27A4 is overexpressed in HCC and acts as a regulator of cell growth, motility and glycolysis induced by sEV‐NAMPT cells. (a)** The relative expression of SLC27A4 in 50 paired non‐tumour and tumour samples from the TCGA‐LIHC cohort *(left)*. K‒M plots showing the disease‐free survival *(middle)* and overall survival *(right)* between patients with high and low SLC27A4 expression (classified by median) in the TCGA‐LIHC cohort. *p* values were obtained by log‐rank tests. **(b)** Immunoblotting confirming the knockdown of SLC27A4 in PLC/PRF/5 cells. β‐Actin was included as an internal control. Colony formation, migration and invasion assays of PLC/PRF/5 CTL‐KD and SLC27A4‐KD cells treated with sEV derived from MHCC97L (97L‐sEV) **(c)** and MHCCLM3 (LM3‐sEV) **(d)** cells. Representative images of colonies and cells are shown. The numbers of colonies and cells were quantified. **(e)** PLC/PRF/5 CTL‐KD and SLC27A4‐KD cells were subcutaneously co‐injected with 97L‐sEV into mice. The tumour volume was monitored regularly. **(f)** A photograph of excised tumours is shown. Tumour weights and dimensions were measured. **(g)** PLC/PRF/5 CTL‐KD and SLC27A4‐KD cells treated with PBS or 97L‐sEV were subjected to ECAR analysis, and the glycoPER was measured. **(h)** PLC/PRF/5 CTL‐KD and SLC27A4‐KD cells treated with PBS, CTL‐SAM‐sEV or NAMPT‐SAM‐sEV were subjected to ECAR and glycoPER measurements. The additions of rotenone/antimycin A (Rot/AA) and 2‐deoxyglucose (2‐DG) are indicated. The data are expressed as the mean ± SEM. ****p < 0.0001, ***p < 0.001, **p < 0.01, *p < 0.05. *NS*, not significant.

Given the high expression of SLC27A4 following sEV‐NAMPT stimulation, we explored its role in HCC development. Analysis of TCGA dataset revealed that a significant number of HCC patients exhibited higher levels of SLC27A4 (*n* = 50) in tumorous tissues than in paired normal tissue samples (Figure 6a). Kaplan–Meier analysis demonstrated strong associations between high SLC27A4 expression and unfavourable disease‐free survival and overall survival (Figure 6a), highlighting the clinical significance of SLC27A4 in HCC. Functionally, SLC27A4‐KD in PLC/PRF/5 and HLE cells impairs cancer properties, as indicated by reduced migration, invasion and colony formation abilities. The carcinogenic responses of the sEV isolated from SLC27A4‐KD cells were also less aggressive (Figures 6b–d and S7a–c). In agreement with the in vitro model, subcutaneous injection of PLC/PRF/5 SLC27A4‐KD cells into mice resulted in significantly slower tumour growth and reduced responsiveness to sEV from metastatic cells compared to CTL‐KD cells (Figure 6e,f).

We further examined the correlation between SLC27A4 levels and glycolytic rate. Treatment of sEV derived from metastatic cells enhanced glycolysis in CTL‐KD cells, but the same treatment failed to activate similar glycolytic promotive effect in SLC27A4‐KD cells (Figures 6g and S7d). Notably, NAMPT‐SAM‐sEVs were unable to induce glycolysis in SLC27A4‐KD cells (Figures 6h and S7e). Collectively, these results emphasize the importance of SLC27A4 as a downstream activator of sEV‐NAMPT and its role in mediating tumour aggressiveness and glycolysis.

### 3.7 Knockdown of SLC27A4 in HCC Significantly Reduced Hepatic TG Levels, Leading to a Decrease in the Glycolytic Rate

**FIGURE 7:**
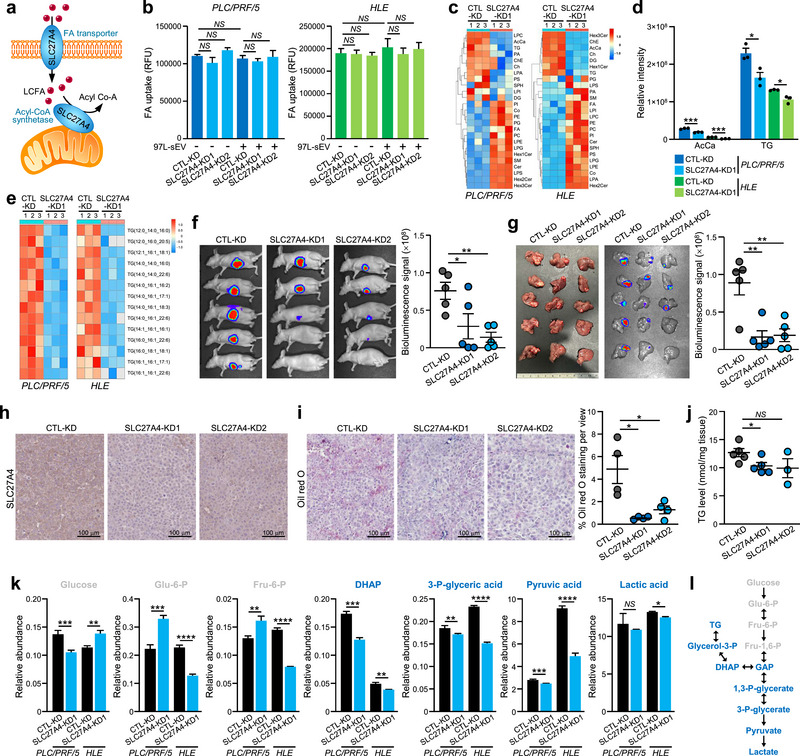
**Knocking down SLC27A4 in HCC significantly reduces hepatic TG levels. (a)** Schematic illustration of the dual role of SLC27A4 as a fatty acid transporter and an acyl‐CoA synthetase. **(b)** Changes in fatty acid (FA) uptake in CTL‐KD and SLC27A4‐KD cells established from PLC/PRF/5 and HLE cells after treatment with 97L‐sEV. **(c)** Heatmap showing the changes in different lipid classes in PLC/PRF/5 and HLE cells after SLC27A4 knockdown. **(d)** Relative level of AcCa and TG in CTL‐KD and SLC27A4‐KD cells. **(e)** Heatmap depicting the changes in TG levels in PLC/PRF/5 and HLE cells after SLC27A4 knockdown. **(f)** The livers of nude mice were orthotopically implanted with PLC/PRF/5 CTL‐KD or SLC27A4‐KD cells. Six weeks after injection, bioluminescence imaging was performed. **(g)** Ex vivo bioluminescence imaging of excised liver tissues. The luciferase signal was quantified. **(h)** Immunohistochemical staining of SLC27A4 in liver tissues. **(i)** Representative micrographs of Oil Red O‐stained (red) liver tumour sections and the quantification of Oil Red O staining are shown. Scale bar, 100 µm. **(j)** TG levels in excised liver tumours were analysed. **(k)** The CTL‐KD and SLC27A4‐KD cells were subjected to metabolite profiling. The quantification of intermediates in CTL‐KD and SLC27A4‐KD cells is presented. **(l)** An illustration of metabolite intermediates involved in the glycolysis pathway. The data are expressed as the mean ± SEM. ****p < 0.0001, ***p < 0.001, **p < 0.01, *p < 0.05, *NS*, not significant.

SLC27A4 mainly functions as a fatty acid transporter for the trafficking of long‐chain fatty acids or as an acyl‐CoA synthetase that catalyses the conversion of unesterified fatty acids to fatty acyl‐CoA (Anderson and Stahl 2013; Grevengoed et al. 2014) (Figure 7a). Interestingly, our findings showed that the loss of SLC27A4 mainly affected the glycolytic rate (Figure 6) but did not result in obvious changes in intracellular fatty acid uptake (Figure 7b). To explore how SLC27A4 mediates glycolysis, we performed lipidomic profiling to delineate its underlying mechanisms. Analysis of lipid classes revealed that SLC27A4 knockdown significantly reduced hepatic acyl carnitine (AcCa) and TG levels in both PLC/PRF/5 and HLE cells (Figure 7c), of which TG existed in higher abundance than AcCa (Figure 7d). Detailed lipidomic further showed a drop in all TG molecules in SLC27A4‐KD cells (Figure 7e). Concurrently, we confirmed the correlation between the TG levels in vivo. In the orthotopic liver implantation model, PLC/PRF/5 SLC27A4‐KD cells exhibited a slower tumour growth rate (Figure 7f,g). Histological analysis of tumour tissues revealed that SLC27A4 levels positively correlated with lipid deposition, as demonstrated by enhanced Oil Red O staining (Figure 7h,i). Moreover, lower TG levels were detected in tumour tissues from the SLC27A4‐KD group than in those from the CTL‐KD group (Figure 7j).

We investigated the relationship between SLC27A4‐mediated increase in TG levels and glycolysis. Metabolomic profiling revealed lower levels of pyruvate and lactate following the reduction in SLC27A4, confirming the involvement of SLC27A4 in glycolysis (Figure 7k). In the early stages of glycolysis, glucose is phosphorylated to glucose‐6‐phosphate (Glu‐6‐P) by hexokinase and subsequently converted to fructose‐6‐phosphate (Fru‐6‐P) (Peeters et al. 2017). Although a reduction in SLC27A4 expression resulted in a decrease in glycolytic end products, we observed that glucose, Glu‐6‐P, and Fru‐6‐P levels were independent of SLC27A4 expression. This finding suggests that SLC27A4‐mediated glycolysis is not initiated by glucose metabolism. Instead, we observed a significant decrease in DHAP levels upon SLC27A4 knockdown (Figure 7k).

DHAP serves as an intermediate metabolite that facilitates the transition between glycolysis and lipogenesis (Hyötyläinen et al. 2016). Under normal cellular conditions, a tightly regulated homeostatic balance is maintained among the various metabolic pathways. In HCC, we observed a positive correlation between SLC27A4 expression and TG levels (Figure 7c). During lipolysis, TG is broken down into fatty acids and glycerol (Quiroga et al. 2018). Glycerol is then phosphorylated by glycerol kinases to form glycerol‐3‐phosphate, which can be further oxidized to DHAP, thereby entering glycolysis (Reshef et al. 2003; Venugopal et al. 2009) (Figure 7l). Therefore, we postulated that SLC27A4 regulates glycolysis through the TG‐induced DHAP transition. Our metabolic profiling supported this hypothesis, as we observed a correlated expression of metabolites beyond the DHAP transition, ranging from DHAP to 3‐phosphoglycerid acid (3‐P‐glyceric acid) (Figure 7l). Collectively, our findings provide new insights into the role of the sEV‐NAMPT/SLC27A4 axis in the regulation of glycolysis.

### 3.8 Therapeutic Potential of Targeting sEV‐NAMPT With the Inhibitor FK866

Based on our findings, we confirmed the role of sEV‐NAMPT in the development of HCC, suggesting the therapeutic potential of targeting sEV‐NAMPT using an inhibitor. We used the well‐studied small molecule inhibitor FK866 and evaluated its efficacy in blocking sEV‐NAMPT. Previous studies have investigated the possibility of using FK866 as a treatment option for liver cancers, including HCC and intrahepatic cholangiocarcinoma (Garten et al. 2017; Pant et al. 2023; Zhang et al. 2018). To validate the therapeutic potential of FK866, we first tested it in a patient‐derived xenograft model with high NAMPT expression and observed that the combined treatment with FK866 and sorafenib had the most potent antitumor effect (Figure S8).

**FIGURE 8:**
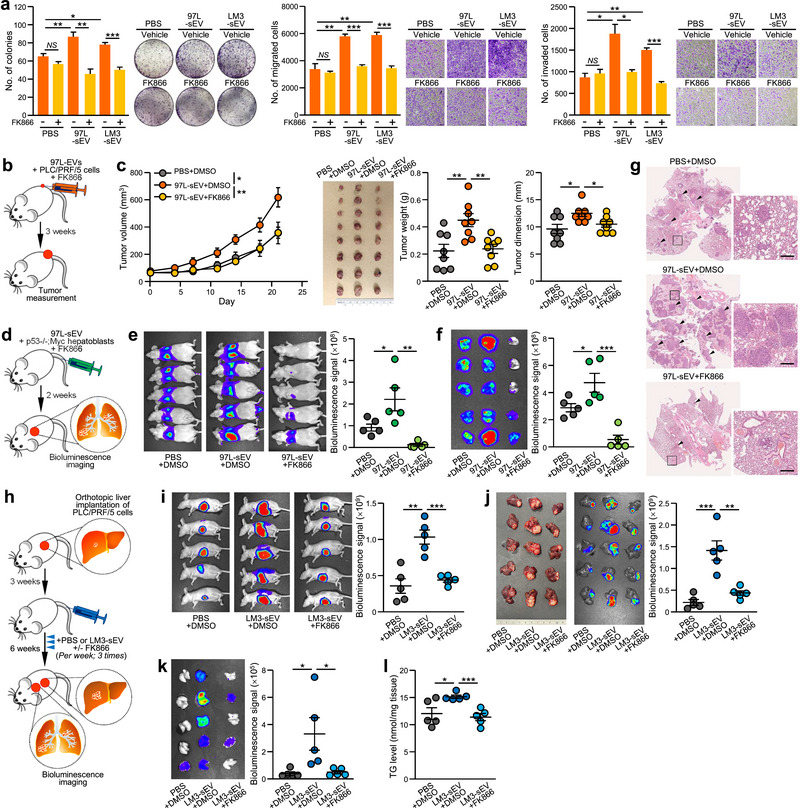
**The NAMPT inhibitor reduces the promoting effect of HCC cell‐derived sEV. (a)** Colony formation, migration and invasion assays of PLC/PRF/5 cells treated with PBS, 97L‐sEV or LM3‐sEV with or without FK866 (2.5 nM) for 24 h. Representative images of colonies and cells are shown. The numbers of colonies and cells were quantified. **(b)** A schematic diagram of the tumour xenograft model in which PLC/PRF/5 cells were co‐injected subcutaneously with 97L‐sEV together with or without FK866 (5 mg/kg mouse body weight). **(c)** Tumour volumes were monitored twice per week. A photograph of the excised tumours is shown. Tumour weights and dimensions were measured. **(d)** A schematic diagram of the lung colonization model. Mice were intravenously injected with murine p53−/−;Myc hepatoblasts with or without 97L‐sEV or FK866 (10 mg/kg mouse body weight). Two weeks after injection, bioluminescence imaging of the animals **(e)** and excised liver tissues **(f)** was performed. The luciferase signal was quantified. **(g)** Representative H&E‐stained micrographs showing tumour nodules in the lungs (indicated by arrowheads). Inlets show enlarged images. Scale bar, 200 µm. **(h)** An illustration of orthotopic liver implantation, sEV education and FK866 treatment. The livers of the mice were implanted with PLC/PRF/5 cells. After 3 weeks, the mice were injected via the tail vein with LM3‐sEV along with DMSO or FK866 (10 mg/kg mouse body weight) once per week for three consecutive weeks. Two weeks after tumour implantation, bioluminescence imaging of the animals **(i)**, excised liver tissues **(j)** and lung tissues **(k)** was performed. The luciferase signal was quantified. **(l)** TG levels in excised liver tumours were analysed. The data are expressed as the mean ± SEM. ***p < 0.001, **p < 0.01, *p < 0.05.

However, most published studies have focused primarily on the activity of FK866 against intracellular NAMPT and its effect on sEV‐NAMPT has not yet been elucidated. Hence, we evaluated the inhibitory effect of FK866 on sEV‐NAMPT in vitro. We observed FK866 only has a limited effect on cells with low NAMPT expression, but could successfully inhibit the promotive effect of sEV derived from MHCC97L and MHCCLM3 cells (Figure 8a). We further extended our evaluation of FK866 expression to various animal models. In these mouse models of HCC performed, treatment with FK866 did not result in significant inhibitory effect on cancer cells in all models (Figure S9). However, the addition of FK866 consistently hindered the oncogenic effects of sEV derived from MHCC97L cells on the growth of subcutaneous tumour derived from PLC/PRF/5 cells (Figure 8b,c) and lung colonization of murine p53−/−;Myc hepatoblasts (Figure 8d,g). To demonstrate that FK866 can inhibit sEV‐NAMPT and its underlying pathway, we used a modified sEV‐education mouse model to mimic our in vitro treatment setting. Orthotopic injection of PLC/PRF/5 cells into the mouse liver was followed by intravenous administration of PBS, MHCCLM3‐sEV, or FK866 2 weeks post‐implantation (Figure 8h). Co‐treatment with FK866 impeded the oncogenic effects of MHCCLM3‐sEV, as indicated by reduced tumour growth and lung metastasis (Figure 8i–k). sEV‐induced TG accumulation in tumour tissues was also inhibited by FK866 (Figure 8l). To confirm the effect of FK866 on inhibiting the promoting ability of LM3‐sEV but not on tumour cells, mouse models of subcutaneous xenograft, lung colonization and orthotopic liver implantation without injection of MHCCLM3‐sEV were treated with FK866 (Figure S9). FK866 treatment on tumours with low NAMPT expression, originating from PLC/PRF/5 cells and murine p53−/−;Myc hepatoblasts, resulted in either no or mild therapeutic effect. TG levels in tumour tissues were also not affected by FK866. Taken together, these in vivo data demonstrated the positive therapeutic effect of FK866 on sEV‐NAMPT.

## 4 Discussion

Cancer cells are known to adopt abnormal metabolic patterns to support their rapid growth and survival in a hostile TME. HCC cells undergo a deregulated metabolic switch characterized by enhanced glycolysis, even in the presence of sufficient oxygen (Shang et al. 2016). In most cases, metabolic reprogramming involves upregulation of glycolytic enzymes and related transporters to facilitate glucose uptake and subsequent metabolic pathways (Amann et al. 2009). These alterations in metabolism play pivotal roles in promoting tumour growth and progression, which are associated with unfavourable prognosis (De Matteis et al. 2018). Hence, it is of paramount importance to uncover the underlying molecular mechanisms and identify the active participants for the development of better therapeutic interventions.

In this study, we investigated the functional role of metastatic HCC‐derived sEV in aggravating aberrant metabolism in the TME. Our data first showed that metastatic HCC‐derived sEV were capable of promoting tumorigenic properties in recipient cells. Subsequent mass spectrometry proteomic and pathway analysis then identified proteins that were upregulated in both metastatic HCC‐sEV were most correlated to the glycolytic pathway. We then mined our data for glycolytic‐related proteins, among all NAMPT is identified as the most predominant upregulated protein in both MHCC97L‐sEV and MHCCLM3‐sEV, prompting a focused investigation into its unexplored functions mediated by sEV in HCC. Despite the fact that we have chosen NAMPT as our target of interest, the presence of other top‐ranked glycolytic‐related proteins, such as MET, has not been overlooked (Boschert et al. 2020). However, since MET's involvement in glycolysis and oncogenesis have been extensively elucidated (Hyung et al. 2023; Peinado et al. 2017), we decided to further our investigation of sEV‐NAMPT.

NAMPT is an essential enzyme involved in the NAD+ salvage pathway, in which it catalyses the conversion of nicotinamide (NAM) to the NAD+ precursor NMN (Garten et al. 2015; Ju et al. 2016). In recent years, eNAMPT and sEV‐NAMPT have attracted increasing attention because of their roles in preventing aging and tumour immune evasion (Imai and Yoshino 2013; Lv et al. 2021). Unlike intracellular NAMPT (iNAMPT), eNAMPT typically lacks enzymatic activity, owing to its limited affinity for substrates (Hara et al. 2011). Therefore, proper internalization into recipient cells is essential for eNAMPT to exert its enzymatic function (Yoshida et al. 2019). In the context of liver cancer, eNAMPT has been shown to regulate immune escape by inducing programmed cell death ligand 1 expression in tumours (Lv et al. 2021). Although the understanding of sEV‐NAMPT in human cancers is currently limited, a study on chronic lymphocytic leukaemia revealed that cancer cells under endoplasmic reticulum stress release NAMPT‐enriched sEV, leading to immunological dysfunction upon interaction with macrophages (Ni et al. 2023). It has also been reported that NAMPT‐containing microvesicles facilitate radioresistance in glioma stem cells and fibroblasts, resulting in unfavourable clinical outcomes (Panizza et al. 2023). TCGA analysis and our in‐house cohort revealed a decrease in NAMPT mRNA and protein levels (Figure S10); however, conversely, we detected an increase in sEV‐NAMPT levels in the serum of HCC patients. This finding suggests that in the context of HCC, the extracellular form of NAMPT, particularly that derived from sEVs, is a major oncogenic mediator. To study the cancerous properties of sEV‐NAMPT, we employed two independent shRNAs to show that the loss of NAMPT hampered the promoting effect of HCC‐sEVs both in vitro and in vivo. We further demonstrated that reduction in NAMPT inhibited the glycolytic effect of HCC‐sEVs. Conversely, overexpression of NAMPT enhanced the oncogenic and glycolytic effects of HCC‐sEVs, further supporting the crucial carcinogenic and glycolytic roles of NAMPT in HCC‐sEVs. Hence, further studies on the underlying mechanisms of action of sEV‐NAMPT in HCC development are warranted.

Interestingly, pathway analysis of sEV‐NAMPT‐treated cells showed that lipid‐related pathways were mainly enhanced instead of glycolysis. We consistently observed an increase in SLC27A4 protein levels in recipient cells following treatment with sEV‐NAMPT. SLC27A4, also known as FATP4, acts as a plasma membrane transporter for long‐chain fatty acids and is abundantly expressed in small intestinal enterocytes (Anderson and Stahl 2013; Ibrahim et al. 2020; Stahl et al. 1999). Analysis of TCGA data revealed that SLC27A4 mRNA levels were significantly increased in HCC and were correlated with poor survival. Previous studies highlighted the aberrant expression and oncogenic role of SLC27A4 in HCC. For example, SLC27A4 promotes the uptake of specific monounsaturated fatty acids (MUFAs), leading to enhanced resistance to ferroptosis through the use of MUFA‐containing phosphatidylcholine (Li et al. 2023). Additionally, dysregulated long non‐coding RNAs in HCC cells upregulate miR‐326, resulting in increased expression of SLC27A4 and contributing to HCC aggressiveness (Ji et al. 2020). The oncogenic role of SLC27A4 in HCC was confirmed in this study, and we further revealed that the loss of SLC27A4 reduced the responsiveness of recipient cells to sEV‐NAMPT. A study reported that treatment with the protective compound torularhodin in a high‐fat diet‐induced mouse model resulted in a marked reduction in SLC27A4 and NAMPT levels (Li et al. 2022), the relationship between these two proteins has not been reported. Our findings show that sEV‐NAMPT regulates SLC27A4 at the transcriptional level via NF‐κB induction. eNAMPT has been widely studied for its ability to regulate various transcription activators, either by direct binding to pattern recognition receptors (PRRs) (Li et al. 2008) or by triggering damage‐associated molecular pattern (DAMP)‐like actions (Tumurkhuu et al. 2023). In addition, eNAMPT can activate NF‐κB signalling by binding to PRRs, namely, TLR4 (Camp et al. 2015), which raises the possibility that sEV‐NAMPT might trigger similar actions. Since NAMPT was present on the surface of the sEV, it was possible for sEV‐NAMPT to interact with TLR4. We further showed that the ability of sEV‐NAMPT to promote tumour growth was dependent on TLR4 expression in recipient cells. However, molecular contact between sEV‐NAMPT and TLR4 was not examined in this study and warrants further investigation.

Given the involvement of SLC27A4 in fatty acid transportation, we hypothesized that the oncogenic properties of SLC27A4 could be attributed to aberrant fatty acid uptake. Surprisingly, depletion of SLC27A4 in HCC cells did not affect intracellular fatty acid levels. To understand this mechanism, we performed a lipidomic analysis and observed a marked reduction in TG levels in SLC27A4‐KD cells. In addition to fatty acid transporters, SLC27A family members can function as acyl‐CoA synthetases (Jia et al. 2007; Liu et al. 2018). Acyl‐CoA synthetase is a critical enzyme involved in lipid metabolism that converts free fatty acids to fatty acyl‐CoA (Wang et al. 2011), which is important for glycerolipid biogenesis and formation. To validate the correlation between SLC27A4 and hepatic TG levels, we used an in vivo orthotopic implantation model and demonstrated that SLC27A4‐KD reduced TG levels in HCC tumours. Conversely, we believe that the upregulation of SLC27A4 induced by sEV‐NAMPT results in the accumulation in liver tumours. Previous studies have reported an abnormal lipid turnover in multiple cancers. For instance, TG degradation was enhanced by the addition of adipose TG lipase (ATGL) for nutrient provision (Yin et al. 2021). Similar lipolytic pathways have also been found in breast cancer and liposarcoma, providing additional energy sources for tumours (Zaidi et al. 2013). In HCC, TG lipolysis was shown to be abnormally upregulated owing to the aberrant expression of ATGL, which drives HCC proliferation (Liu et al. 2018). Moreover, abnormal lipid recycling is further aggravated by enhanced fat deposition (Gong et al. 2014; Saponaro et al. 2015). Therefore, we hypothesized that SLC27A4‐mediated TG overload triggered additional lipid turnover. This speculation was confirmed by the increased production of DHAP, a breakdown product of TG to glycerol, which enters glycolysis. This speculation is consistent with our metabolomic data, which showed an increase in the glycolytic pathway after DHAP. These findings highlight a previously unrecognized mechanism of SLC27A4‐mediated glycolysis, which was not induced by the conventional starting metabolite glucose but was instead triggered by the transition of DHAP from the lipolysis pathway.

Finally, we demonstrated the therapeutic potential of FK866, a competitive inhibitor of NAMPT (Fratta et al. 2023; Hasmann and Schemainda 2003), in blocking sEV‐NAMPT as a treatment option in various animal models. In addition, FK866 inhibited the sEV‐NAMPT‐SLC27A4 pathway because a reduced TG concentration was observed in the treatment group. These findings suggest the possibility of targeting sEV‐NAMPT as a promising therapeutic strategy for HCC patients. Although FK866 had a positive outcome in our study, we are aware that phase II clinical trials of cutaneous T‐cell lymphoma have failed because of its high toxicity (Wei and Zhang 2022). With the development of better NAMPT inhibitors, such as antibody‐drug conjugates and more potent inhibitory compounds, further studies are needed to determine the optimal inhibitory compound.

In summary, our study provides valuable insights into the underlying mechanism by which sEV‐NAMPT promotes HCC aggressiveness and enhances glycolysis through SLC27A4 and NF‐κB transactivation via TLR4. These findings have significant implications, as they shed light on the potential of sEV‐NAMPT as a biomarker for HCC and a promising therapeutic target.

## Author Contributions

**Cherlie Lot Sum Yeung**: Data curation (equal); formal analysis (equal); investigation (equal); validation (equal); writing‐original draft (equal); writing–review and editing (equal). **Tung Him Ng**: Data curation (equal); formal analysis (equal); investigation (equal); validation (equal); writing‐original draft (equal); writing–review and editing (supporting). **Charlotte Jiaqi Lai**: Investigation (equal). **Tingmao Xue**: Investigation (supporting). **Xiaowen Mao**: Methodology (equal). **Sze Keong Tey**: Methodology (equal). **Regina Cheuk Lam Lo**: Formal analysis (supporting). **Chun‐Fung Sin**: Formal analysis (supporting); resources (supporting). **Kwan Ming Ng**: Methodology (supporting). **Danny Ka Ho Wong**: Resources (supporting). **Lung‐yi Mak**: Resources (supporting). **Man‐Fung Yuen**: Resources (supporting). **Irene Oi‐Lin Ng**: Resources (supporting). **Peihua Cao**: Resources (supporting). **Yi Gao**: Resources (lead). **Jing Ping Yun**: Resources (supporting). **Judy Wai Ping Yam**: Conceptualization (lead); funding acquisition (lead); project administration (lead); supervision (lead); writing–review and editing (lead).

## Conflicts of Interest

The authors declare no conflicts of interest.

## Supporting information



Supporting Information

## Data Availability

Data available on request from the authors.
