# Supplementary material for: Small Extracellular Vesicle‐Derived Nicotinamide Phosphoribosyltransferase (NAMPT) Induces Acyl‐Coenzyme A Synthetase SLC27A4‐Mediated Glycolysis to Promote Hepatocellular Carcinoma
Source: J Extracell Vesicles. 2025 Apr 16;14(4):e70071. doi: 10.1002/jev2.70071 (PMC12000932; doi:10.1002/jev2.70071)
Supplement: Supplementary file 1 — Supporting Information [file JEV2-14-e70071-s001.docx]

**Small extracellular vesicle-derived nicotinamide phosphoribosyltransferase (NAMPT) induces acyl coenzyme A synthetase SLC27A4-mediated glycolysis to promote hepatocellular carcinoma**

Cherlie Lot Sum Yeung^1,#^, Tung Him Ng^1,#^, Charlotte Jiaqi Lai^1^, Tingmao Xue^1,2^, Xiaowen Mao^3^, Sze Keong Tey^4^, Regina Cheuk Lam Lo^1^, Chun-Fung Sin^1^, Kwan Ming Ng^5^, Danny Ka Ho Wong^6^, Lung-Yi Mak^6,7^, Man-Fung Yuen^6,7^, Irene Oi-Lin Ng^1,7^, Peihua Cao^8^, Yi Gao^2^, Jing Ping Yun^9^, Judy Wai Ping Yam^1,7,10,^*

^1^Department of Pathology, Li Ka Shing Faculty of Medicine, The University of Hong Kong, Hong Kong

^2^Department of Hepatobiliary Surgery II, Zhujiang Hospital, Southern Medical University, Guangzhou, Guangdong, China

^3^State Key Laboratory of Quality Research in Chinese Medicine, Institute of Chinese Medical Sciences, University of Macau, Macau

^4^Department of Surgery, School of Clinical Medicine, Li Ka Shing Faculty of Medicine, The University of Hong Kong, Hong Kong

^5^Laboratory for Synthetic Chemistry and Chemical Biology Limited, Hong Kong Science Park, Hong Kong

^6^Department of Medicine, Li Ka Shing Faculty of Medicine, The University of Hong Kong, Hong Kong

^7^State Key Laboratory of Liver Research (The University of Hong Kong), Hong Kong

^8^Clinical Research Center, Zhujiang Hospital, Southern Medical University, Guangzhou, Guangdong, China

^9^Department of Pathology, Sun Yat-sen University Cancer Center, Guangzhou, Guangdong, China

^10^Materials Innovation Institute for Life Sciences and Energy (MILES), HKU-SIRI, Shenzhen, China

^#^These authors contributed equally

**Supplementary Materials and Methods**

**Protein extraction and Western blotting analysis**

Proteins were extracted by homogenizing cells or sEV in radioimmunoprecipitation assay buffer supplemented with 10% PhosSTOP™ Phosphatase Inhibitor Cocktail (Roche) and 10% cOmplete™ Protease Inhibitor Cocktail (Roche) at 4°C for 30 minutes. Insoluble debris were removed by centrifugation at 13,000×g for 20 minutes. Isolated proteins were then mixed with SDS loading buffer, resolved by SDS-polyacrylamide gel electrophoresis, and transferred onto a polyvinylidene difluoride (PVDF) membrane (GE Healthcare). The membrane was sequentially incubated with primary antibodies at 4°C overnight and horseradish peroxidase (HRP)-conjugated secondary antibodies at ambient temperature for 1 hour. Chemiluminescence was developed using HRP substrate (GE Healthcare) and visualized under Amersham Imager 680 (GE Healthcare).

**Enzyme-linked immunosorbent assay (ELISA)**

The level of NAMPT in sEV were measured using Human NAMPT ELISA Kit (FineTest) following supplier’s suggested protocol. In brief, sEV were diluted in the provided sample dilution buffer, and then added to the ELISA microplate and incubated for 90 minutes. After incubation, microplate was washed and subsequently incubated with biotin-labelled antibody for 60 minutes and then with HRP-streptavidin conjugate for 30 minutes. Colorimetric signals were developed with tetramethylbenzidine substrate and absorbance was measured at 450 nm using Infinite F200 microplate reader (Tecan). Sample NAMPT levels were deduced from the standard curve generated using the provided NAMPT standard.

**RNA extraction, reverse transcription and quantitative polymerase chain reaction**

Total RNA was extracted using TRIzol™ reagent as per manufacturer’s instructions. Extracted RNA was quantified using BioDrop spectrophotometer. One microgram of RNA was reverse transcribed into cDNA using SuperScript™ VILO™ Master Mix following supplier’s protocol. An aliquot of cDNA was amplified by quantitative PCR using PowerUp™ SYBR™ Green Master Mix on LightCycler® 480 System (Roche) (Cycling condition: 95°C for two minutes, followed by 40 cycles of 95°C for 15 second, 60°C for 15 second and 72°C for one minute). Relative expression of target genes was calculated and normalized against HPRT expression using 2^-ΔΔCt^ method. Primer sequences were listed in Table S2.

**Real-time cell metabolic analysis**

Extracellular acidification rate (ECAR) was measured to estimate cell glycolytic rate using Seahorse XF96 Extracellular Flux Analyzer (Agilent) following supplier’s protocol. Briefly, treated cells were seeded onto Seahorse XF96 cell culture microplate and allowed to settle overnight. Prior to assay, culture medium was replaced with fresh Seahorse XF DMEM medium (supplemented with 100 mM glucose, 20 mM glutamine and 10 mM pyruvate). ECAR was then measured using default Glycolytic Rate Assay protocol included in Wave software (Agilent). In details, ECAR was measured 3 times. Subsequently, 0.5 μM Rotenone and Antimycin A were added immediately after the third measurement. Measurements were performed for 3 more times. After the sixth measurement, 50 mM 2-deoxyglucose was added, followed by 5 more measurements. Each measurement was performed for 3 minutes, separated by a 2-minute mixing interval. A total of 11 measurements were performed. At the end of assay, proton efflux rate (glycoPER) was calculated to represent cell glycolytic rate according to manufacturer’s guidelines.

**Colony formation assay**

Treated cells were seeded in 6-well plate in triplicate at a density of 1,000 cells per well. After incubation for 1 to 2 weeks, cells were fixed in methanol and stained with crystal violet. Colonies formed were counted.

**Cell migration and invasion assays**

Both assays were performed using Transwell® inserts (polycarbonate membrane with 8µm pore size) (Corning) based on supplier’s protocol. For migration assay, treated cells were suspended in serum-free medium and added into the upper chamber. Culture medium supplemented with 10% FBS and hepatocyte growth factor (20 ng/mL) were added to the lower chambers as chemoattractant. Cells were allowed to migrate for 16-18 hours. After incubation, unmigrated cells were removed using a cotton swab. Migrated cells were fixed with methanol, stained with crystal violet and imaged under a microscope. Cells were counted in 4 random fields in each insert. Experiments were carried out in triplicate. For invasion assay, procedures were the same except that inserts were coated with a layer of Matrigel® Matrix Basement Medium (Corning).

**Immunofluorescence**

Cells were allowed to settle on cover glass overnight and treated with sEV. Treated cells were then fixed in 4% paraformaldehyde for 10 minutes, permeabilized in 0.1% triton X-100 for 10 minutes and blocked in 3% BSA in PBS for 2 hours. The cells were then incubated with antibodies targeting NF-κB (CST), followed by Alexa Fluor 488-conjugated secondary antibodies. Cells were counterstained in 1 μg/mL 4′,6-diamidino-2-phenylindole (DAPI) for 10 minutes and imaged under LSM900 confocal microscope (Zeiss).

**Fatty acid uptake**

Treated cells were seeded at a density of 10,000 cells per well onto 96-well plate. After attachment, the cells were washed with PBS and incubated with 2 µM BODIPY-C_12_ (Thermo Fisher) in dark at 37°C for 4 minutes. After incubation, cells were washed with PBS and treated with 0.08% trypan blue to quench extracellular fluorescence. Intracellular fluorescence was measured at Ex/Em = 488/520 nm using Infinite F200 microplate reader (Tecan).

**Measurement of triacylglycerol level**

Cells or tumor tissues were homogenized in 5% NP-40 (USB) in water. The lysates were heated to 95°C for 5 minutes and cooled to ambient temperature. The heating was repeated for a total of 2 cycles. Then, the mixture was centrifuged at 13,200 rpm for 2 minutes. Triglyceride in the supernatant was quantified using Triglyceride Assay Kit (Abcam) following supplier’s protocol. Measured triglyceride levels were normalized based on cell number or tissue weight.

**Animal husbandry and ethical issues**

BALB/cAnN-nu (Nude) mice were housed in the Centre for Comparative Medicine Research (CCMR) at the University, ensuring a controlled, pathogen-free environment with adequate air conditioning. The experimental procedures adhered to the guidelines set forth by the animal ethics committee of the University, as outlined in the approved research protocol CULATR 5531-20 and 6007-22. All procedures were conducted in accordance with the Animals (Control of Experiments) Ordinance, which is regulated by the Department of Health in Hong Kong.

**Lung colonization study**

Hundred thousand luciferase-labelled mouse p53-/-;Myc hepatoblasts together with 10 μg sEV or FK866 (10 mg/kg mouse body weight) or vehicle were injected intravenously through tail vein into 7-week-old male mice. After 2 weeks, mice were anesthetized and injected intraperitoneally with luciferin (150 mg/kg body weight). Bioluminescence signal from the lung of the mice was measured *in vivo* and *ex vivo* using IVIS Spectrum *in vivo* imagining system (Perkin Elmer). Mice were sacrificed and lungs were collected for histological analysis.

**Tumor xenograft model**

One and half million naïve or stably transfected PLC/PRF/5 cells were suspended in Matrigel® Matrix and co-injected subcutaneously with 10 μg sEV or FK866 (5mg/kg mouse body weight) or vehicle into the flank of five-week-old male mice. Tumor size was monitored and measured using a caliper at indicated time points throughout experiments. At the end of experiments, mice were sacrificed. Tumors were excised, weighted and examined. Tumor volume was defined as ½ × length × width^2^. Tumor dimension was defined as the largest diameter of the tumors.

**Orthotopic liver implantation**

One million of luciferase-labelled cells were suspended in 15 μL Matrigel® Matrix and orthotopically injected into the left lobe of the liver in each 6-week-old male mouse under anesthesia. Six weeks after injection, mice were subjected to bioluminescence imaging using IVIS Spectrum *in vivo* imagining system. Mice were sacrificed and livers were collected for biochemical and histological analysis.

**Immunohistochemical study**

Formalin-fixed paraffin-embedded tissue microarray or tumor tissues were sectioned at 5 μm thickness, deparaffinized and rehydrated. Antigens were retrieved in sodium citrate buffer (10 mM sodium citrate, 0.05% Tween 20, pH 6.0) at 98°C for 20 minutes. Then, endogenous peroxidase activity was blocked in 3% hydrogen peroxide for 10 minutes. Sections were incubated with 10% normal goat serum (Dako) and then with primary antibodies against NAMPT (LSBio) or SLC27A4 (Abcam) followed by biotinylated secondary antibodies and HRP-conjugated streptavidin. Chromogen development was performed using 3,3’-diaminobenzidine tetrahydrochloride. Finally, slides were counterstained with hematoxylin, and scanned under Hamamatsu NanoZoomer S210 (Japan). Expression was examined by an experienced pathologist without prior knowledge in case information.

**The Cancer Genome Atlas (TCGA) database analysis**

Expression data of 50 pairs of tumor and non-tumor tissues were collected from TCGA-LIHC data directory. Reads mapped to NAMPT and SLC27A4 were used to quantify mRNA expression of NAMPT and SLC27A4 respectively. Expressions were normalized and expressed in binary logarithmic scale.

**Mass spectrometric analysis**

Protein was extracted by digesting cells or sEV in lysis buffer (8 M urea in 100 mM tris, pH 8.5) at 4^o^C for 60 minutes. Lysate was then centrifuged at 13,000×g for 20 minutes to remove insoluble debris. Twenty micrograms of protein were reduced and alkylated in tris(2-carboxyethyl) phosphine and iodoacetamide respectively. Mixture was then diluted 10-fold in 100 mM ammonium bicarbonate. Trypsin was added at 1:50 ratio (trypsin:protein) and incubated at 37°C for 16 hours. Digestion was quenched in 5% formic acid. The peptides were desalted using C18 STAGE tip. Purified peptides were concentrated and reconstituted in 20 µL of 0.1% formic acid for LC-MS/MS analysis. One microliter of peptides was separated using a C18 column (50 cm×75 µm, 1.9 µm pore size; Thermo Fisher) on a nanoLC system coupled to an Orbitrap Fusion™ Lumos™ Tribrid™ Mass Spectrometer (Thermo Fisher). The mobile phase consisted of (A) 0.1% formic acid and (B) 80% acetonitrile in 0.1% formic acid, with B running from 5% to 80% linearly for 150 minutes at a flow rate of 150 nL/minute. Mass spectrometer was operated in a data-dependent acquisition mode cycling through full MS1 scan (resolution: 120,000; scan range: 350-1,500*m/z*; AGC target: 2×10^6^; maximum ion injection time: 50 ms), followed by HCD MS2 scan (isolation window: 1.6*m/z*; normalized collision energy: 30; resolution: 30,000; AGC target: 1×10^5^; maximum ion injection time: 100 ms) every 10 MS/MS spectra. Raw data were searched against the Uniprot Human database using Proteome Discoverer Software (Thermo Fisher).

**Profiling of polar metabolites and lipids**

Sample processing and analysis were performed at Centre for PanorOmic Sciences - Proteomics and Metabolomics Core, LKS Faculty of Medicine, The University of Hong Kong. For metabolite profiling, cell pellets (five million cells) were washed with ice-cold saline and then extracted in 2 mL of 80% ice-cold methanol containing 0.02 mg/L norvaline (internal standard) in dry ice. The lysates were then centrifuged at 14,000×g for 20 minutes at 4°C for protein precipitation. One milliliter of supernatant was dried under a gentle stream of nitrogen at room temperature. The dried aliquots were then resuspended with 500 μL 50% acetonitrile, vortexed for 10 seconds and centrifuged for two minutes at 14,000×g. The supernatant was dried under nitrogen. The dried residues were redissolved and derivatized for two hours at 37°C in 40 μL of methoxylamine hydrochloride (30 mg/mL in pyridine) followed by trimethylsilylation for one hour at 37°C in 70 μL MSTFA with 1% TMCS. Up to 1 μL sample was injected for gas chromatography-tandem mass spectrometry (GC-MS/MS) analysis. Chromatogram was acquired in SCAN and MRM mode in an Agilent 7890B GC - Agilent 7010 Triple Quadrapole Mass Spectrometer system (Santa Clara, CA, USA). The sample was separated through an Agilent (Santa Clara, CA, USA) DB-5MS capillary column (30 m×0.25 mm ID, 0.25 µm film thickness) under constant flow of helium at one milliliter per minute. The GC oven program started at 60°C (hold time 1 minute) and was increased 10°C/minute to 120°C, then 3°C/minute to 150°C, and then 10°C/minute to 200°C and finally 30°C/minute to 280°C (hold 5 minutes). Inlet temperature and transfer line temperature were 250°C and 280°C respectively. Characteristic quantifier and qualifier transitions were monitored in MRM mode during the run. Mass spectra from *m/z* 50-500 were acquired in SCAN mode. Data analysis was performed using the Agilent MassHunter Workstation Quantitative Analysis Software. Linear calibration curves for each analyte were generated by plotting peak area ratio of external/internal standard against standard concentration at different concentration levels. Analytes were confirmed by comparing the retention time and ratio of characteristic transitions between the sample and standard.

For lipid profiling, cell pellets (5 million cells) were washed with ice-cold saline and then extracted in 5 mL of chloroform: methanol (2:1, v/v). The samples were sonicated for 20 seconds twice, separated by a 10-second interval, and then centrifuged at 3,000×g for five minutes. Supernatant of 1.5 mL was aliquoted and dried under nitrogen. The dried residuals were reconstituted in 50 μL isopropanol: methanol: chloroform (1:1:0.2, v/v). Sample were injected for liquid chromatography-tandem mass spectrometry (LC-MS/MS) analysis. Chromatographic separation was carried out in Accucore C30 HPLC column (150×2.1 mm, 2.6 μm); Thermo Fisher) on an Vanquish UPLC system (Thermo Fisher, Waltham, MA, USA). Mobile phases were (A) 10 mM ammonium formate with 0.1% formic acid in acetonitrile and water (6:4, v/v) and (B) 10 mM ammonium formate with 0.1% formic acid in acetonitrile and isopropanol (1:9, v/v). Three microliters sample was separated at a flow rate of 0.26 mL/minute. Column temperature was set at 45°C. The gradient started at 30% B and was increased to 43% B in 2 minutes, then increased to 55% B in 2.1 minutes, 65% B in 12 minutes, 85% B in 18 minutes and 100% B in 20 minutes (held for 5 minutes), and decreased linearly to 30% B for column re-equilibration. Mass spectrometry analysis was processed using an Orbitrap Exploris 120 mass spectrometer (Thermo Fisher, Waltham, MA, USA) equipped with a HESI II probe in polar switching mode with source parameters set as follows: sheath gas flow rate: 60; auxiliary gas flow rate: 17; sweep gas flow rate: 1; spray voltage: +3.5/-3.0 kV; capillary temperature: 275°C; S-lens RF level: 70; and heater temperature: 325°C. Data was collected at dd-MS2 mode. Data analysis was performed using Lipidsearch (Thermo Fisher/Mitsui Knowledge Industries) with the default parameters for Orbitrap MS Product Search and Alignment. After alignment, raw peak areas for all identified lipids were extracted.

**Supplementary Table**

**Supplementary Table S1. Information of serum donors**

| **Clinical parameters** | **Category** | **Number of donors** |
| --- | --- | --- |
| **HCC patients** |  |  |
| Gender | Male | 43 |
|  | Female | 10 |
| Age | ≤ 60 | 28 |
|  | > 60 | 25 |
| HBsAg* | Positive | 33 |
|  | Negative | 11 |
| Cirrhotic liver* | Cirrhosis | 22 |
|  | Normal and CH | 22 |
| pTMN stage | I and II | 32 |
|  | III and IV | 21 |
| **HCC patients before and after surgery** | | |
| Gender | Male | 15 |
|  | Female | 4 |
| Age | ≤ 60 | 10 |
|  | > 60 | 9 |
| pTMN stage | I and II | 15 |
|  | III and IV | 4 |
| **Individuals with chronic HBV infection and cirrhosis** | | |
| Gender | Male | 29 |
|  | Female | 0 |
| Age | ≤ 60 | 9 |
|  | > 60 | 20 |
| **Control individuals** |  |  |
| Gender | Male | 15 |
|  | Female | 15 |
| Age | < 60 | 12 |
|  | > 60 | 18 |

HBsAg, Hepatitis B surface antigen; CH, Chronic hepatitis; pTNM = Pathological tumor-node-metastasis; ALT, alanine aminotransferase; AST, aspartate aminotransferase; AFP, alpha fetal protein

*Missing information in some cases

**Supplementary Table S2. Sequences of oligos used in this study**

| **Primer name** | **Oligo sequence 5′ to 3′** |
| --- | --- |
| shNAMPT-1F | CCGGCCACCTTATCTTAGAGTTATTCTCGAGAATAACTCTAAGATAAGGTGGTTTTT |
| shNAMPT-1R | AATTCAAAAACCACCTTATCTTAGAGTTATTCTCGAGAATAACTCTAAGATAAGGTG |
| shNAMPT-2F | CCGGGTAACTTAGATGGTCTGGAATCTCGAGATTCCAGACCATCTAAGTTACTTTTT |
| shNAMPT-2R | AATTCAAAAAGTAACTTAGATGGTCTGGAATCTCGAGATTCCAGACCATCTAAGTTA |
| shSLC27A4-1F | CCGGCCGGGTCTTCATCAAGACCATCTCGAGATGGTCTTGATGAAGACCCGGTTTTTG |
| shSLC27A4-1R | AATTCAAAAACCGGGTCTTCATCAAGACCATCTCGAGATGGTCTTGATGAAGACCCGG |
| shSLC27A4-2F | CCGGCTTCACAGATAAACTGTTCTACTCGAGTAGAACAGTTTATCTGTGAAGTTTTTG |
| shSLC27A4-2R | AATTCAAAAACTTCACAGATAAACTGTTCTACTCGAGTAGAACAGTTTATCTGTGAAG |
| shTLR4-1F | CCGGGCCACCTCTCTACCTTAATATCTCGAGATATTAAGGTAGAGAGGTGGCTTTTTG |
| shTLR4-1R | AATTCAAAAAGCCACCTCTCTACCTTAATATCTCGAGATATTAAGGTAGAGAGGTGGC |
| shTLR4-2F | CCGGCCCTGCTGGATGGTAAATCATCTCGAGATGATTTACCATCCAGCAGGGTTTTTG |
| shTLR4-2R | AATTCAAAAACCCTGCTGGATGGTAAATCATCTCGAGATGATTTACCATCCAGCAGGG |
| sgNAMPT-1F | CACCGAAGCGCCCGGGTCACGCGCC |
| sgNAMPT-1R | AAACGGCGCGTGACCCGGGCGCTTC |
| sgNAMPT-3F | CACCGCTAAGTTCGAGTTCCCGGCA |
| sgNAMPT-3R | AAACTGCCGGGAACTCGAACTTAGC |
| NAMPT-F | AATGTTCTCTTCACGGTGGAAAA |
| NAMPT-R | ACTGTGATTGGATACCAGGACT |
| SLC27A4-F | GGACCCAGGTGGGATTCTC |
| SLC27A4-R | CGCGCCTGATGGTCTTGAT |
| HRPT1-F | CTTTGCTGACCTGCTGGATT |
| HRPT1-R | CTGCATTGTTTTGCCAGTGT |

**Supplementary Table S3. Proteins upregulated by sEV-NAMPT in HLE cells**

| **Protein**  **name** | **Log2**  **(CTL-KD-sEV/**  **NAMPT-KD1-sEV)** | **Log2**  **(CTL-KD-sEV**  **/PBS)** |  | **Protein**  **name** | | **Log2**  **(CTL-KD-sEV/**  **NAMPT-KD1-sEV)** | **Log2**  **(CTL-KD-sEV**  **/PBS)** |
| --- | --- | --- | --- | --- | --- | --- | --- |
| CYBC1 | 3.342 | 3.915 |  | RETSAT | | 2.213 | 1.800 |
| PPP3CB | 2.835 | 1.394 |  | DHODH | | 2.211 | 1.218 |
| ARMC10 | 2.652 | 2.211 |  | CYP20A1 | | 2.210 | 2.183 |
| SLC25A20 | 2.639 | 1.807 |  | C15orf24 | | 2.193 | 1.705 |
| ZMPSTE24 | 2.634 | 2.025 |  | SRPRA | | 2.193 | 1.786 |
| **SLC27A4** | **2.611** | **2.264** |  | CCDC47 | | 2.191 | 1.610 |
| NDC1 | 2.608 | 2.328 |  | ALG5 | | 2.187 | 1.531 |
| MOSPD1 | 2.596 | 2.288 |  | OSBPL8 | | 2.181 | 1.793 |
| FNDC3B | 2.586 | 2.126 |  | CISD2 | | 2.164 | 1.717 |
| HACD3 | 2.560 | 1.791 |  | CERS2 | | 2.161 | 1.746 |
| LPCAT1 | 2.547 | 2.055 |  | NDUFAF4 | | 2.161 | 1.233 |
| WFS1 | 2.547 | 2.167 |  | TMPO | | 2.155 | 1.840 |
| DAD1 | 2.544 | 1.958 |  | H3-2 | | 2.154 | 1.534 |
| MTP18 | 2.541 | 1.017 |  | ASPH | | 2.151 | 1.864 |
| MPDU1 | 2.523 | 1.873 |  | ZNF638 | | 2.151 | 1.709 |
| VRK2 | 2.489 | 2.131 |  | DHRS7 | | 2.151 | 1.851 |
| PGRMC1 | 2.453 | 1.818 |  | CTCF | | 2.150 | 1.827 |
| CAMLG | 2.447 | 2.283 |  | CLPTM1L | | 2.150 | 1.671 |
| MGST1 | 2.447 | 1.776 |  | EPHX1 | | 2.149 | 1.605 |
| AMFR | 2.415 | 1.652 |  | LPCAT4 | | 2.149 | 2.161 |
| PNPLA6 | 2.414 | 1.763 |  | HMGN1 | | 2.145 | 1.672 |
| SEC61A1 | 2.408 | 1.754 |  | SACM1L | | 2.139 | 1.937 |
| ZNF512 | 2.405 | 2.227 |  | TMEM214 | | 2.136 | 1.725 |
| SMPD4 | 2.384 | 1.665 |  | LPCAT2 | | 2.133 | 1.963 |
| SHINC3 | 2.368 | 1.786 |  | TMEM205 | | 2.132 | 1.582 |
| FADS1 | 2.343 | 1.780 |  | LNPK | | 2.127 | 1.558 |
| MKI67 | 2.341 | 1.830 |  | SGPL1 | | 2.124 | 1.785 |
| MMGT1 | 2.332 | 2.268 |  | HBA2 | | 2.123 | 1.677 |
| RFC1 | 2.327 | 1.838 |  | MLEC | | 2.120 | 1.736 |
| SEC63 | 2.323 | 1.900 |  | HIST1H4J | | 2.118 | 1.420 |
| FAM241A | 2.319 | 2.059 |  | DIDO1 | | 2.101 | 1.386 |
| LRRC59 | 2.302 | 1.917 |  | EMD | | 2.087 | 1.674 |
| TMX2 | 2.296 | 1.989 |  | PTRH2 | | 2.082 | 1.641 |
| C10orf70 | 2.294 | 1.116 |  | BAZ1B | | 2.081 | 1.373 |
| TMCO1 | 2.291 | 1.815 |  | EMC3 | | 2.080 | 1.573 |
| PREB | 2.286 | 2.107 |  | SRPRB | | 2.070 | 1.679 |
| GCS1 | 2.270 | 1.597 |  | CYP51A1 | | 2.065 | 1.455 |
| VAPB | 2.263 | 1.737 |  | MTCH1 | | 2.062 | 1.235 |
| RDH11 | 2.258 | 1.671 |  | MAVS | | 2.059 | 1.039 |
| THEM6 | 2.257 | 1.676 |  | TOR1AIP1 | | 2.059 | 1.391 |
| UNC84A | 2.250 | 1.913 |  | TOP2A | | 2.057 | 1.453 |
| FNDC3A | 2.247 | 1.847 |  | SEL1L | | 2.055 | 1.478 |
| STBD1 | 2.242 | 1.316 |  | MARCHF5 | | 2.050 | 1.206 |
| HMOX1 | 2.236 | 1.447 |  | CANX | | 2.047 | 1.571 |
| **Protein**  **name** | **Log2**  **(CTL-KD-sEV/**  **NAMPT-KD1-sEV)** | **Log2**  **(CTL-KD-sEV**  **/PBS)** |  | **Protein**  **name** | **Log2**  **(CTL-KD-sEV/**  **NAMPT-KD1-sEV)** | | **Log2**  **(CTL-KD-sEV**  **/PBS)** |
| PTPN1 | 2.045 | 1.060 |  | ATP5MK | 1.825 | | 1.048 |
| H3-3A/-3B | 2.031 | 1.495 |  | PBXIP1 | 1.823 | | 1.425 |
| NCEH1 | 2.027 | 1.468 |  | APOOL | 1.818 | | 1.456 |
| TMEM43 | 2.022 | 1.621 |  | C10orf35 | 1.813 | | 1.357 |
| DPM1 | 2.022 | 1.233 |  | GOSR2 | 1.812 | | 1.337 |
| Nbla03646 | 2.021 | 1.630 |  | STX18 | 1.799 | | 1.207 |
| ATL3 | 2.015 | 1.499 |  | TMUB1 | 1.793 | | 1.138 |
| COMT | 2.012 | 1.451 |  | KIF22 | 1.793 | | 1.102 |
| GK | 2.012 | 1.196 |  | DDX21 | 1.780 | | 1.170 |
| PRAF2 | 2.008 | 1.538 |  | CYB5B | 1.774 | | 1.460 |
| DLGAP5 | 1.999 | 2.083 |  | TOMM70 | 1.774 | | 1.160 |
| VDAC3 | 1.991 | 1.137 |  | RPRC1 | 1.773 | | 1.386 |
| NDUFA13 | 1.978 | 1.359 |  | CHD4 | 1.773 | | 1.167 |
| NOA1 | 1.976 | 1.103 |  | THOC2 | 1.769 | | 1.200 |
| SEC11A | 1.974 | 1.096 |  | YIF1B | 1.769 | | 1.782 |
| TMX1 | 1.973 | 1.574 |  | KTN1 | 1.767 | | 1.357 |
| UQCRQ | 1.971 | 1.412 |  | STX5 | 1.766 | | 1.280 |
| MTCH2 | 1.966 | 1.099 |  | RPN2 | 1.766 | | 1.184 |
| DNMT1 | 1.964 | 1.367 |  | HCTP4 | 1.757 | | 1.149 |
| APMAP | 1.956 | 1.386 |  | TMED10 | 1.753 | | 1.110 |
| ATP2A2 | 1.953 | 1.463 |  | FAM62A | 1.748 | | 1.117 |
| SPCS2 | 1.951 | 1.670 |  | DRG2 | 1.748 | | 1.187 |
| COX2 | 1.943 | 1.053 |  | SCCPDH | 1.745 | | 1.095 |
| DDOST | 1.938 | 1.337 |  | MRPL43 | 1.743 | | 1.111 |
| TOMM40 | 1.934 | 1.005 |  | KIF23 | 1.742 | | 1.396 |
| H1-5 | 1.932 | 1.219 |  | ATL2 | 1.739 | | 1.110 |
| SSR1 | 1.932 | 1.626 |  | UTP20 | 1.733 | | 1.080 |
| TMX3 | 1.929 | 1.458 |  | TKT | 1.732 | | 1.409 |
| TMEM167A | 1.929 | 1.356 |  | DHRS7B | 1.727 | | 1.232 |
| RPN1 | 1.916 | 1.418 |  | LETM1 | 1.718 | | 1.014 |
| CYB5R3 | 1.900 | 1.408 |  | MICAL2 | 1.716 | | 1.221 |
| ABHD12 | 1.891 | 1.262 |  | NUSAP1 | 1.708 | | 1.066 |
| TOMM6 | 1.887 | 1.345 |  | RRBP1 | 1.706 | | 1.527 |
| ESYT2 | 1.886 | 1.409 |  | ALDH3A2 | 1.691 | | 1.114 |
| MAP1B | 1.880 | 1.553 |  | IER3IP1 | 1.678 | | 1.359 |
| NDUFB8 | 1.880 | 1.372 |  | LBR | 1.672 | | 1.186 |
| CKAP5 | 1.878 | 1.621 |  | C7orf50 | 1.669 | | 1.170 |
| FAF2 | 1.875 | 1.225 |  | SMARCA5 | 1.658 | | 1.005 |
| MGST3 | 1.869 | 1.016 |  | IFI16 | 1.655 | | 1.331 |
| SEC22B | 1.865 | 1.391 |  | PARP1 | 1.654 | | 1.244 |
| ERLIN1 | 1.854 | 1.432 |  | AKR1C1 | 1.652 | | 2.767 |
| AFG3L2 | 1.850 | 1.018 |  | RACGAP1 | 1.640 | | 1.246 |
| HELLS | 1.848 | 1.446 |  | DDX52 | 1.626 | | 1.214 |
| COX20 | 1.842 | 1.085 |  | MRPL14 | 1.615 | | 1.114 |
| TOP2B | 1.839 | 1.221 |  | TRIP12 | 1.595 | | 1.130 |
| ERGIC1 | 1.831 | 1.367 |  | ARMCX1 | 1.591 | | 1.035 |
| **Protein**  **name** | **Log2**  **(CTL-KD-sEV/**  **NAMPT-KD1-sEV)** | **Log2**  **(CTL-KD-sEV**  **/PBS)** |  | **Protein**  **name** | **Log2**  **(CTL-KD-sEV/**  **NAMPT-KD1-sEV)** | | **Log2**  **(CTL-KD-sEV**  **/PBS)** |
| STK10 | 1.576 | 1.111 |  | MTDH | 1.413 | | 1.366 |
| SPIN | 1.576 | 1.426 |  | CEP131 | 1.373 | | 1.020 |
| TOMM20 | 1.561 | 1.127 |  | MAP4 | 1.337 | | 1.364 |
| PRKDC | 1.547 | 1.063 |  | SERBP1 | 1.324 | | 1.111 |
| PDS5A | 1.469 | 1.003 |  | SEC61B | 1.079 | | 1.377 |
| LEMD3 | 1.446 | 1.057 |  | TMEM256-PLSCR3 | 1.073 | | 1.180 |

**Supplementary Figure**


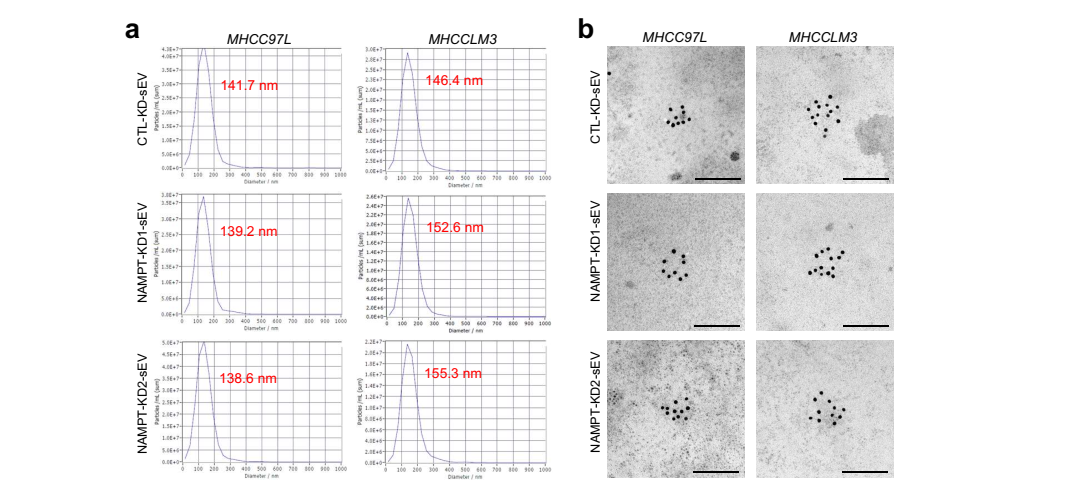


**Supplementary Fig 1. Characterization of sEV derived from MHCC97L and MHCCLM3 cells upon NAMPT knockdown. (a)** Size distribution of the indicated sEV measured by ZetaView® Nanoparticle Tracking Analyzer (Particle Metrix GmbH). **(b)** Representative electron micrographs of the indicated sEV after immunogold labeling. sEV were labeled by anti-CD63 antibodies followed by secondary antibodies coupled to 10-nm gold particles. Scale bar, 100 nm; Magnification, 52,000×.


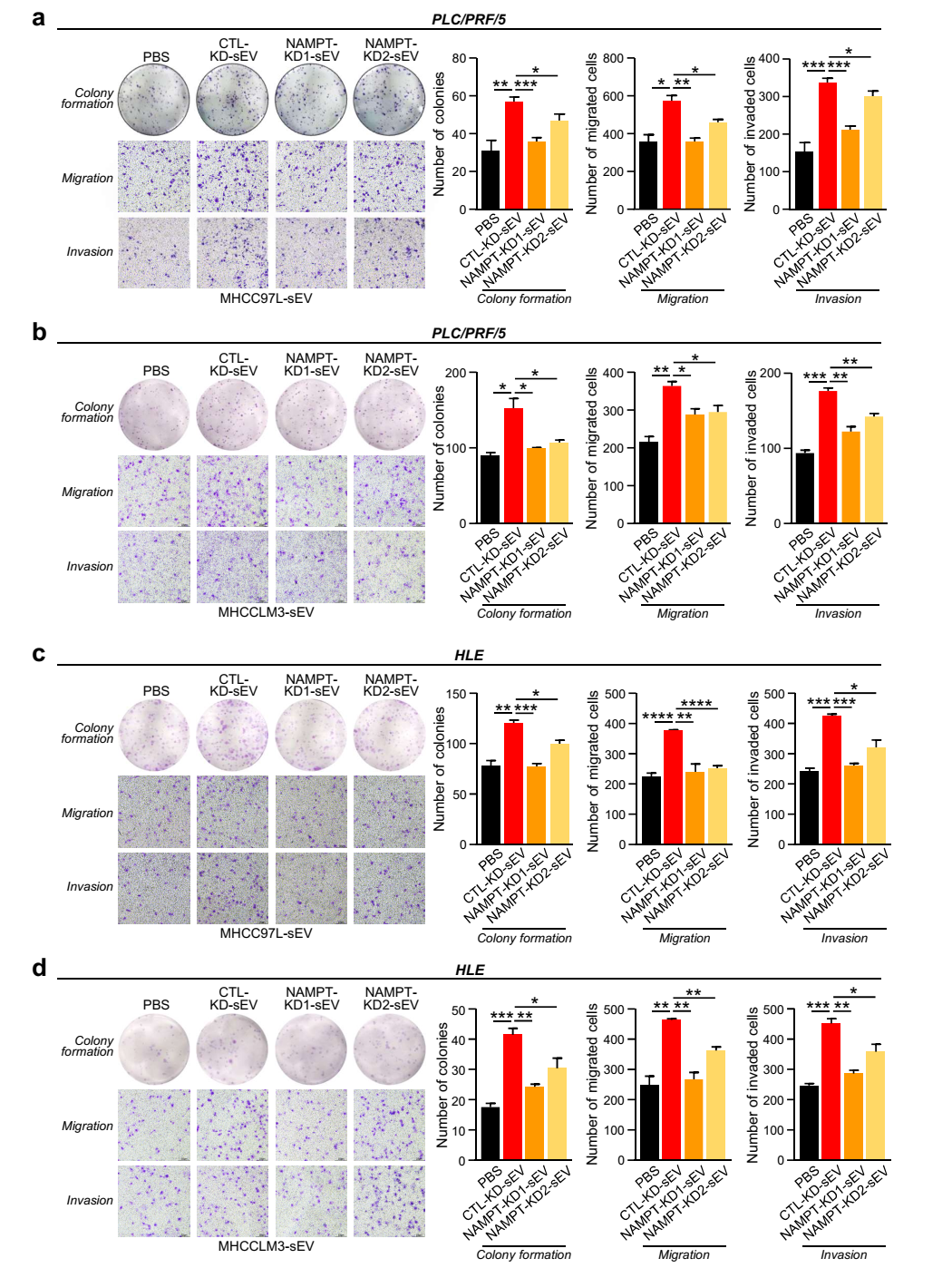


**Supplementary Fig 2. NAMPT-knockdown in sEV derived from metastatic HCC cells showed reduced potential to promote HCC cancerous phenotypes.** Colony formation, migration and invasion assays for PLC/PRF/5 cells treated with the indicated sEV derived from MHCC97L **(a)** and MHCCLM3 **(b)** cells, and HLE cells treated with the sEV from MHCC97L **(c)** and MHCCLM3 **(d)** cells. Representative images of colonies and cells are shown. Number of colonies and number of migrated and invaded cells were counted. Data are expressed as mean ± SEM. *****P* < 0.0001, ****P* < 0.001, ***P* < 0.01, **P* < 0.05.


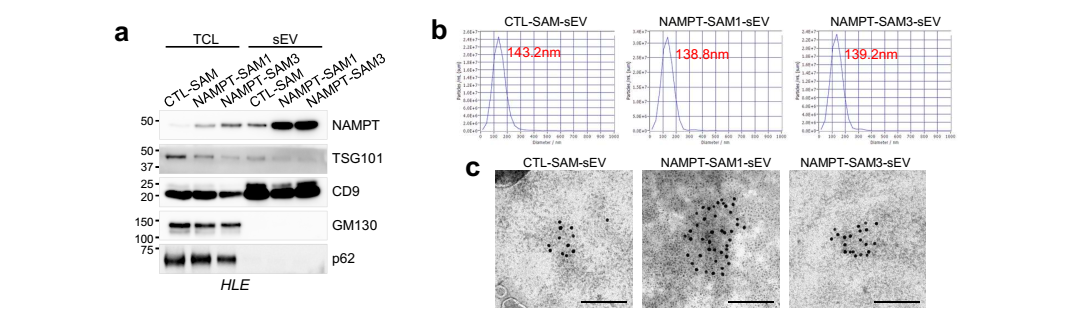


**Supplementary Fig 3. Characterization of sEV derived from HLE cells after NAMPT overexpression. (a)** Immunoblotting for NAMPT, positive (TSG101, CD9) and negative (*cis*-Golgi marker GM130, nucleoporin p62) sEV markers in the total cell lysate (TCL) and sEV of HLE CTL-SAM and NAMPT-SAM cells. NAMPT activation was confirmed and sEV identity was validated. **(b)** Size distributions of the indicated sEV measured by ZetaView® Nanoparticle Tracking Analyzer (Particle Metrix GmbH). **(c)** Representative electron micrographs of the indicated sEV after immunogold labeling. sEV were labeled by anti-CD63 antibodies followed by secondary antibodies coupled to 10-nm gold particles. Scale bar, 100 nm; Magnification, 52,000×.


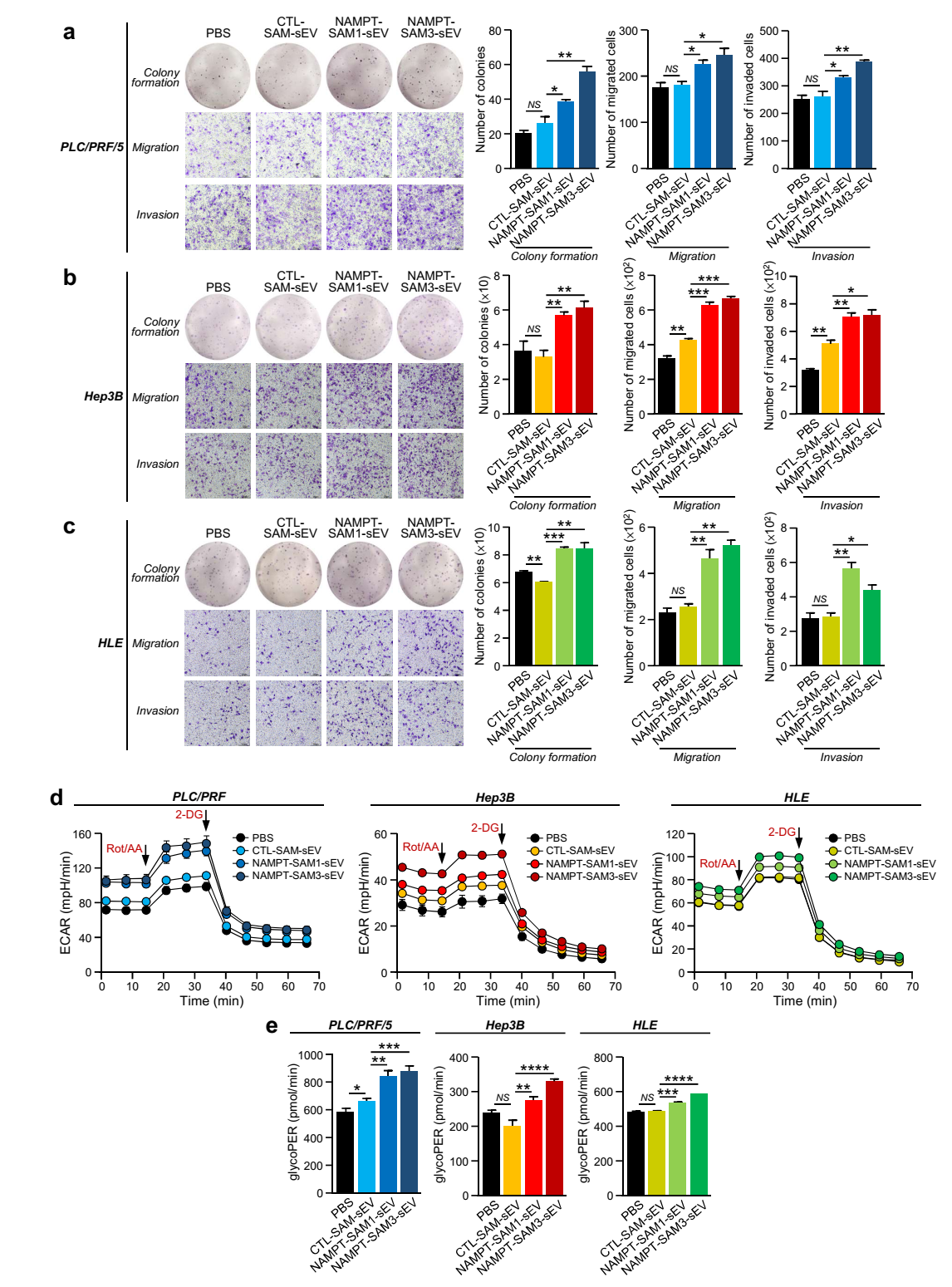


**Supplementary Fig 4. sEV-derived NAMPT facilitated HCC progression and cell glycolysis. (a-c)** Colony formation, migration and invasion assay for PLC/PRF/5 **(a)**, Hep3B **(b)** and HLE **(c)** cells treated with sEV derived from HLE CTL-SAM and NAMPT-SAM cells. Representative images of colonies and cells are shown. Number of colonies and number of migrated and invaded cells were counted. **(d)** Seahorse glycolytic rate assay to monitor real-time changes in ECAR for HCC cells treated with indicated sEV. **(e)** glycoPER was measured. Addition of Rotenone/Antimycin A (Rot/AA) and 2-deoxyglucose (2-DG) are indicated. Data are expressed as mean ± SEM. *****P* < 0.0001, ****P* < 0.001, ***P* < 0.01, **P* < 0.05. *NS*, not significant.


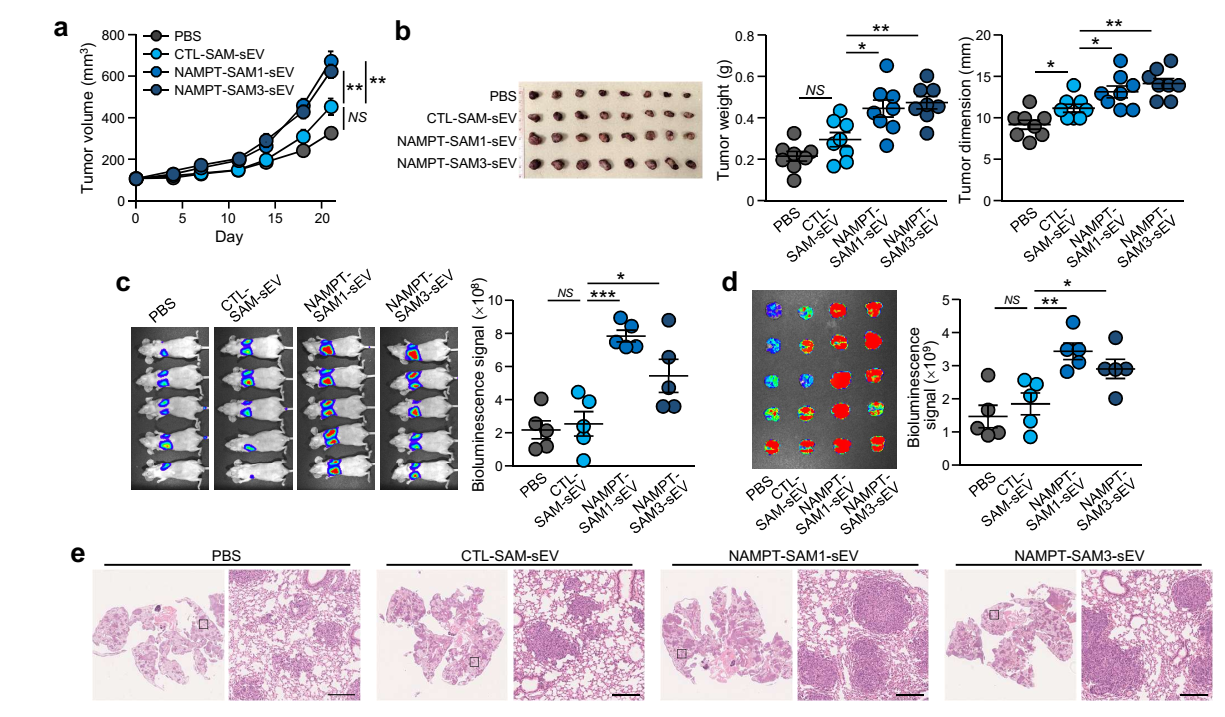


**Supplementary Fig 5. sEV-derived NAMPT promoted HCC progression and metastasis *in vivo*. (a)** PLC/PRF/5 cells were co-injected with sEV derived from HLE CTL-SAM and NAMPT-SAM cells subcutaneously into the flank of nude mice. Tumor volumes were monitored twice per week. **(b)** Photograph of excised tumors is shown. Tumor weight and dimension were measured. **(c-e)** Murine p53-/-;Myc hepatoblast together with indicated sEV were intravenously injected into nude mice. After two weeks, bioluminescence imaging of live animal **(c)** and excised lung tissues **(d)** were performed. Luciferase signal was quantified. **(e)** Representative H&E-stained micrographs showing tumor nodules in lungs. Enlarged images are shown. Scale bar, 200 µm. Data are expressed as mean ± SEM. ****P* < 0.001, ***P* < 0.01, **P* < 0.05. *NS*, not significant.


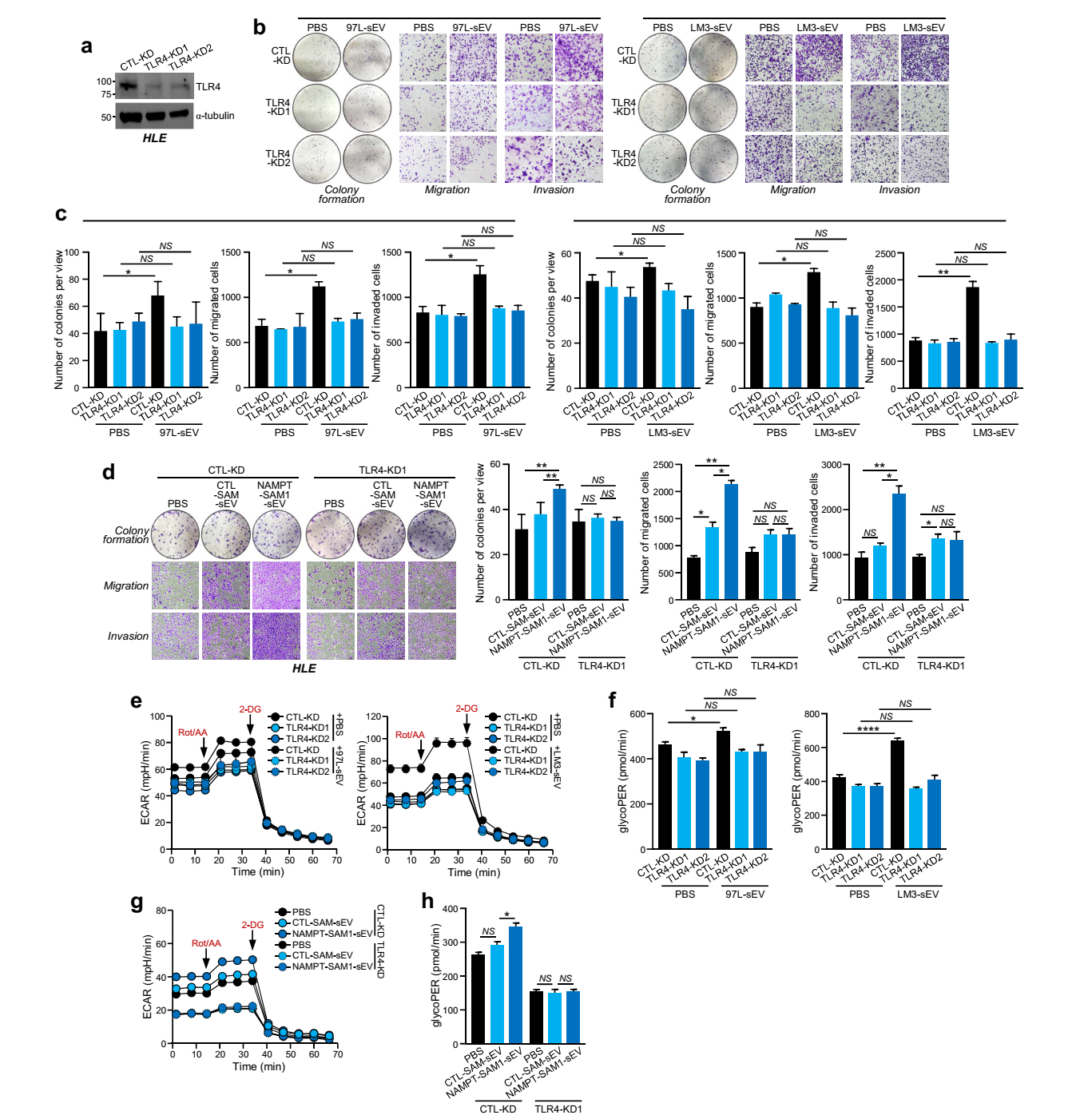


**Supplementary Fig 6. TLR4 knockdown abolishes cancer promoting effects of metastatic HCC cells-derived sEV. (a)** Immunoblotting of TLR4 in CTL-KD and TLR4-KD cells established in HLE cells. α-tubulin was used as an internal control. **(b)** HLE CTL-KD and TLR4-KD cells treated with sEV derived from MHCC97L (97L-sEV) and MHCCLM3 (LM3-sEV) cells were subjected to colony formation, migration and invasion assays. Representative images of colonies and cells are shown. **(c)** Number of colonies and cells were quantified. **(d)** HLE CTL-KD and TLR4-KD cells treated with PBS, CTL-SAM-sEV or NAMPT-SAM-sEV were subjected to migration, invasion and colony formation assays. Representative images of colonies and cells are shown. Number of colonies and cells were counted. **(e and f)** Seahorse glycolytic rate assay for HLE CTL-KD and TLR4-KD cells treated with sEV derived from MHCC97L and MHCCLM3 cells. Real-time changes in ECAR **(e)** and the measured glycoPER **(f)** are shown. **(g and h)** Seahorse glycolytic rate assay for HLE CTL-KD and TLR4-KD cells treated with HLE CTL-SAM and NAMPT-SAM1 sEV. Real-time changes in ECAR **(g)** and the measured glycoPER **(h)** are shown. Addition of Rotenone/Antimycin A (Rot/AA) and 2-deoxyglucose (2-DG) are indicated. Data are expressed as mean ± SEM. *****P* < 0.0001, ***P* < 0.01, **P* < 0.05. *NS*, not significant.


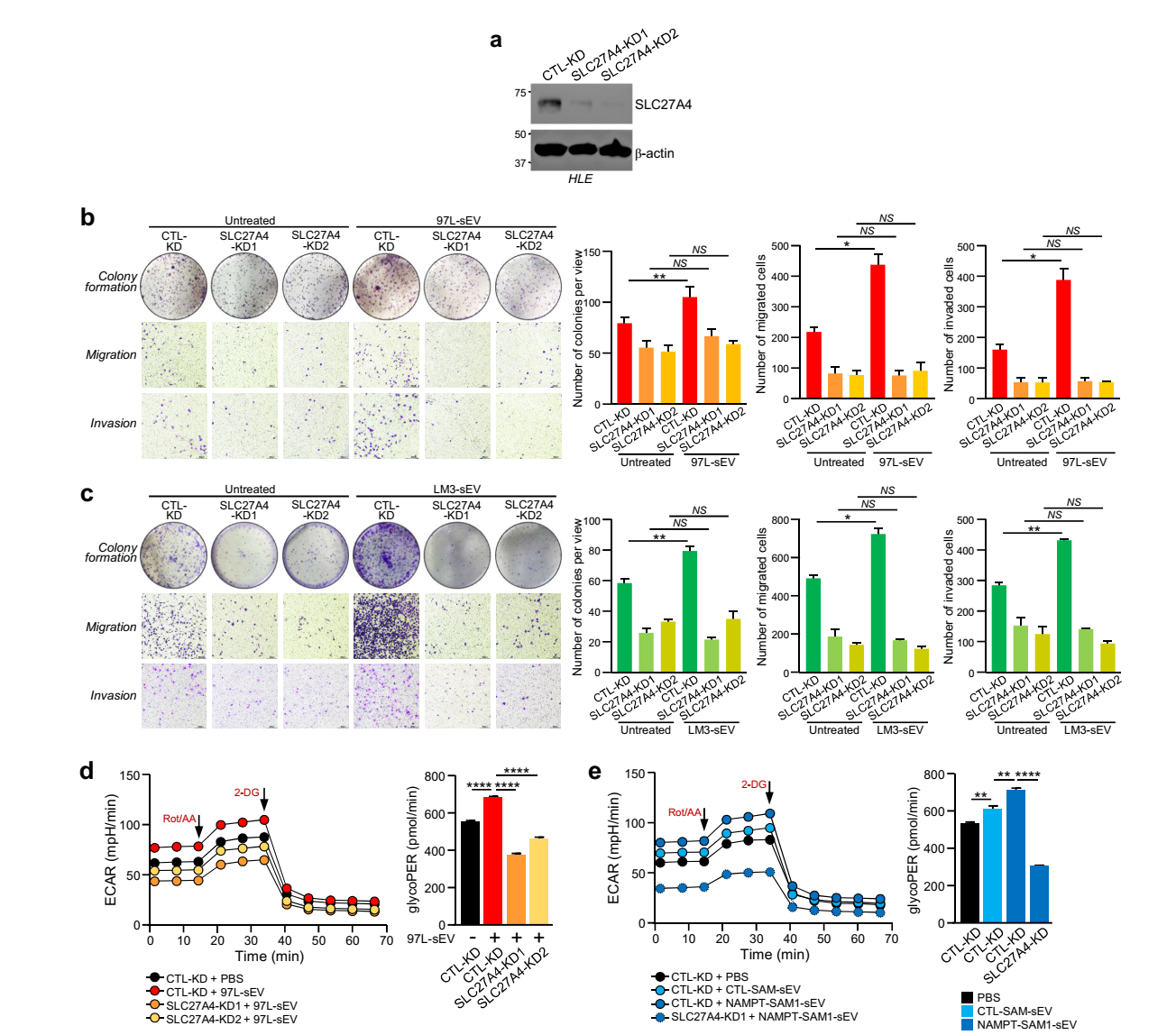


**Supplementary Fig 7. Knockdown of SLC27A4 abolished HCC cell-derived sEV-enhanced cancer properties and glycolysis. (a)** Immunoblotting of SLC27A4 in CTL-KD and SLC27A4-KD cells established in HLE cells. β-actin was used as an internal control. HLE CTL-KD and SLC27A4-KD cells were treated with sEVs derived from MHCC97L cells **(b)** and MHCCLM3 cells **(c)** were subjected to migration, invasion and colony formation assay. Representative images of colonies and cells are shown. Number of colonies and number of migrated and invaded cells were quantified. **(d and e)** Seahorse glycolytic rate assay for CTL-KD and SLC27A4-KD cells treated with MHCC97L-sEV **(d)** and HLE NAMPT-SAM1-sEV **(e)**. Real-time changes in ECAR and measured glycoPER are shown. Addition of Rotenone/Antimycin A (Rot/AA) and 2-deoxyglucose (2-DG) are indicated. Data are expressed as mean ± SEM. *****P* < 0.0001, ***P* < 0.01, **P* < 0.05. *NS*, not significant.


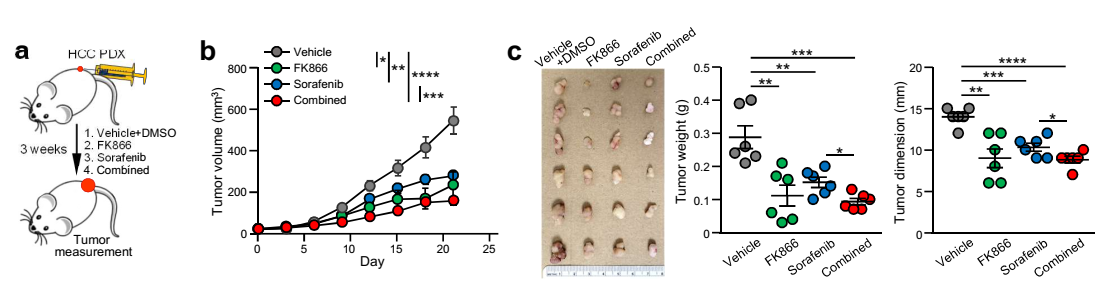


**Supplementary Fig 8. Co-treatment effects of FK866 and sorafenib on patient-derived xenograft model. (a)** Schematic illustration of patient-derived xenograft (PDX) model. Mice were treated as indicated for 3 weeks. **(b)** Tumor volumes were monitored twice per week. **(c)** Photograph of excised tumors was shown. Tumor weight and dimension were measured. Data are expressed as mean ± SEM. *****P* < 0.0001, ****P* < 0.001, ***P* < 0.01, **P* < 0.05.


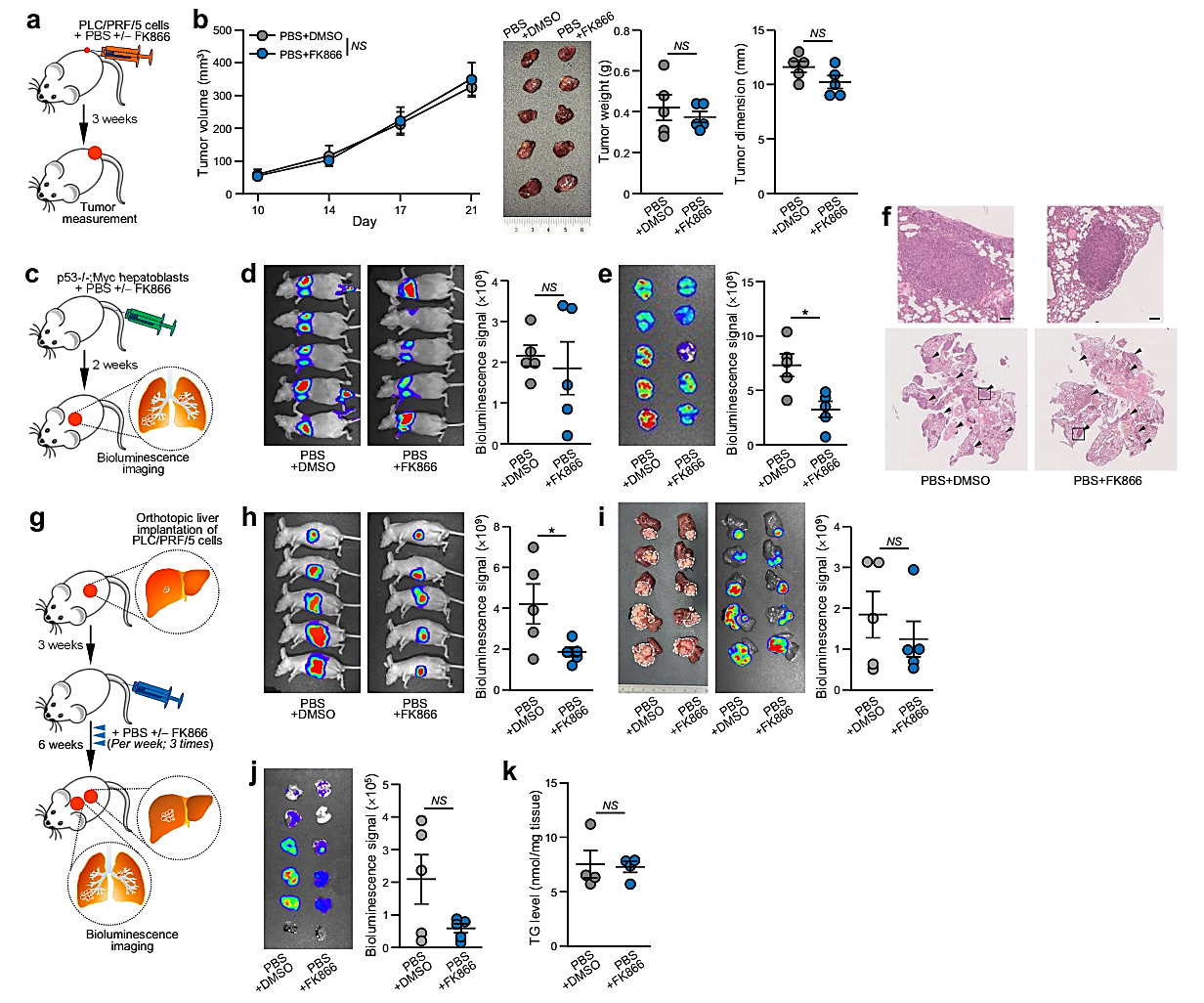


**Supplementary Fig 9. Effect of NAMPT inhibitor FK866 on PLC/PRF/5 cells with low expression of NAMPT. (a)** A schematic diagram of the tumor xenograft model in which PLC/PRF/5 cells were coinjected subcutaneously with or without FK866 (5 mg/kg mouse body weight). **(b)** Tumor volumes were monitored for 3 weeks. A photograph of the excised tumors is shown. Tumor weights and dimensions were measured. **(c)** A schematic diagram of the lung colonization model. Mice were intravenously injected with murine p53-/-;Myc hepatoblasts with or without FK866 (10 mg/kg mouse body weight). Two weeks after injection, bioluminescence imaging of the animals **(d)** and excised lung tissues **(e)** was performed. The luciferase signal was quantified. **(f)** Representative H&E-stained micrographs showing tumor nodules in the lungs (indicated by arrowheads). Inlets show enlarged images. Scale bar, 100 µm. **(g)** An illustration of orthotopic liver implantation of PLC/PRF/5 cells. After three weeks, the mice were injected via the tail vein with DMSO or FK866 (10 mg/kg mouse body weight) once per week for three consecutive weeks. Two weeks after tumor implantation, bioluminescence imaging of the animals **(h)**, excised liver tissues **(i)** and lung tissues **(j)** was performed. The luciferase signal was quantified. **(k)** TG levels in excised liver tumors were analyzed. The data are expressed as the mean ± SEM. **P* < 0.05. *NS*, not significant.


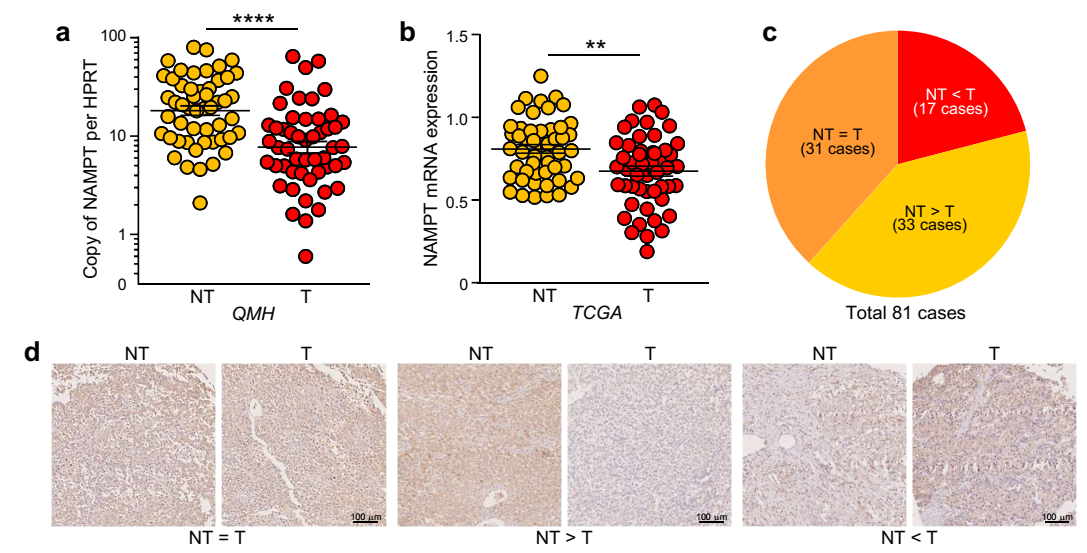


**Supplementary Fig 9. NAMPT expression in HCC.** (**a)** Quantitative PCR analysis to compare NAMPT expression between non-tumor (NT) and tumor (T) tissues in 51 pairs of HCC clinical samples from Queen Mary Hospital (QMH) cohort. **(b)** NAMPT expression in 50 paired NT and T cases from TCGA-LIHC cohort. **(c)** Distribution of NAMPT overexpression (NT < T), no change (NT = T) and underexpression (NT > T) cases determined by immunohistochemical staining of NAMPT in tissue microarray, and **(d)** representative micrographs of respective cases. Data are expressed as mean ± SEM. *****P* < 0.0001, ***P* < 0.01.
